# UHRF1 regulates AR ubiquitination to promote the loss of AR signaling and enzalutamide resistance in progression of prostate cancer

**DOI:** 10.1038/s41419-026-08511-9

**Published:** 2026-02-27

**Authors:** Yifan Zhang, Zhaojun Yu, Yadong Li, Mayao Luo, Wenli Hou, Sangsang Li, Jiapeng He, Shidong Lv, Qiang Wei, Hailiang Hu

**Affiliations:** 1 Department of Urology, Guangdong Cardiovascular Institute, Guangdong Provincial People’s Hospital, Guangdong Academy of Medical Sciences, Southern Medical University, Guangzhou, China; 2 Department of Biochemistry, SUSTech Homeostatic Medicine Institute, School of Medicine, Southern University of Science and Technology, Shenzhen, China; 3 Department of Urology, Nanfang Hospital, Southern Medical University, Guangzhou, China; 4 Postdoctoral Innovation Practice Base, The First Affiliated Hospital, Jiangxi Medical College, Nanchang University, Nanchang, China; 5 Department of Urology, Ganzhou Hospital-Nanfang Hospital, Southern Medical University, Ganzhou, Jiangxi China

**Keywords:** Prostate cancer, Cancer therapeutic resistance

## Abstract

Lineage plasticity has emerged as an important mechanism of treatment resistance in prostate cancer, increasingly associated with loss of androgen receptor (AR) signaling, and in many cases induction of stemness phenotypes and neuroendocrine features. However, targeted therapies for this stage of the disease are currently lacking. In this study, we demonstrated the critical role of the epigenetic regulator UHRF1 in the enzalutamide resistance development of prostate cancer. We have shown that UHRF1 is highly expressed in enzalutamide-resistant prostate cancer cells and its expression correlates with the loss of AR-dependent glandular features. Knocking down UHRF1 led to increased AR expression and enhanced the activity of canonical AR signaling pathway in prostate cancer cells. The combination of UHRF1 knockdown with enzalutamide treatment demonstrated synergistic tumor inhibitory effects both in vitro and in vivo. Mechanistically, UHRF1 was found to bind to AR and promote its ubiquitination and degradation. Furthermore, inhibition of UHRF1 restored AR pathway activity and re-sensitized resistant prostate cancer cells to enzalutamide. Therefore, our findings elucidate an intracellular molecular mechanism that promotes prostate cancer lineage plasticity and suggest that UHRF1 may serve as a potential therapeutic target for overcoming resistance to AR-targeted therapies.

## Introduction

Androgen receptor (AR), a critical driver of prostate cancer (PCa) development, is the main target for anti-androgen therapy of PCa patients, including androgen-deprivation therapy and the second generation of AR pathway inhibitors (ARPI) such as enzalutamide [1]. However, through an AR-independent mechanism whereby luminal-type PCa transdifferentiates into a mixture of multilineage PCa, such as treatment-induced neuroendocrine prostate cancer (t-NEPC), double-negative prostate cancer and stem-like cancer, that are AR-indifferent disease and no longer sensitive to AR signaling blockades [2]. Although AR is thought to be absent in primary NEPC patients, more than a quarter of the t-NEPC patients expresses AR but with inactivated classical AR signaling [3–5]. It has been demonstrated that EZH2 inhibition reverses the lineage plasticity of t-NEPC and restores tumor sensitivity to ARPIs [6]; however, late-stage AR-negative NEPC organoids are unable to undergo lineage switch [7], suggestive of the existence of a transitional state between the AR-positive and AR-negative NEPC [8]. Currently, no effective therapies exist to target lineage plasticity–driven resistance in tumors, emphasizing the urgent need to identify key modifiers of lineage plasticity.

In addition to genomic changes, non-mutational epigenetic reprogramming has been identified as a crucial factor in cellular lineage decisions and therapeutic responses in PCa [9]. UHRF1 is a multi-domain multifunctional nuclear protein with a major role in the maintenance of DNA methylation [10]. The RING domain of UHRF1 exhibits E3 ubiquitin ligase activity, which catalyzes the monoubiquitylation of lysine 18 and 23 at the N-terminus of histone H3 (H3K18Ub, H3K23Ub) to generate DNMT1 binding sites [11]. UHRF1 also catalyzes the polyubiquitination of non-histone substrates such as EG5, thereby promoting their degradation through the ubiquitin-proteasome pathway [12]. In PCa, UHRF1 deficiency reactivates tumor suppressor genes by affecting epigenetic features, such as histone H3K9 methylation, acetylation, and DNA methylation [13]. Additional studies have demonstrated that UHRF1 functions as the “gatekeeper” of the AMPK pathway and is regulated by the transcription factor ASCL1, which participates in the lineage remodeling process in PCa [5, 14]. However, whether UHRF1 influences the lineage plasticity of PCa and the regulatory mechanism of UHRF1 in the AR pathway remains elusive.

In this study, we demonstrated that upregulation of UHRF1 in PCa results in inactivation of classical AR signaling. UHRF1 facilitates AR polyubiquitination and degradation, consequently driving cancer cells from an AR-dependent luminal lineage to a state of lineage characterized by the expression of stemness and neuroendocrine (NE)-like biomarkers. These lineages are AR-independent and unresponsive to AR-targeted therapies such as enzalutamide. Notably, the suppression of UHRF1 re-establishes tumor sensitivity to AR-targeted therapies.

## Results

### UHRF1 expression increases with PCa progression

**Fig. 1:**
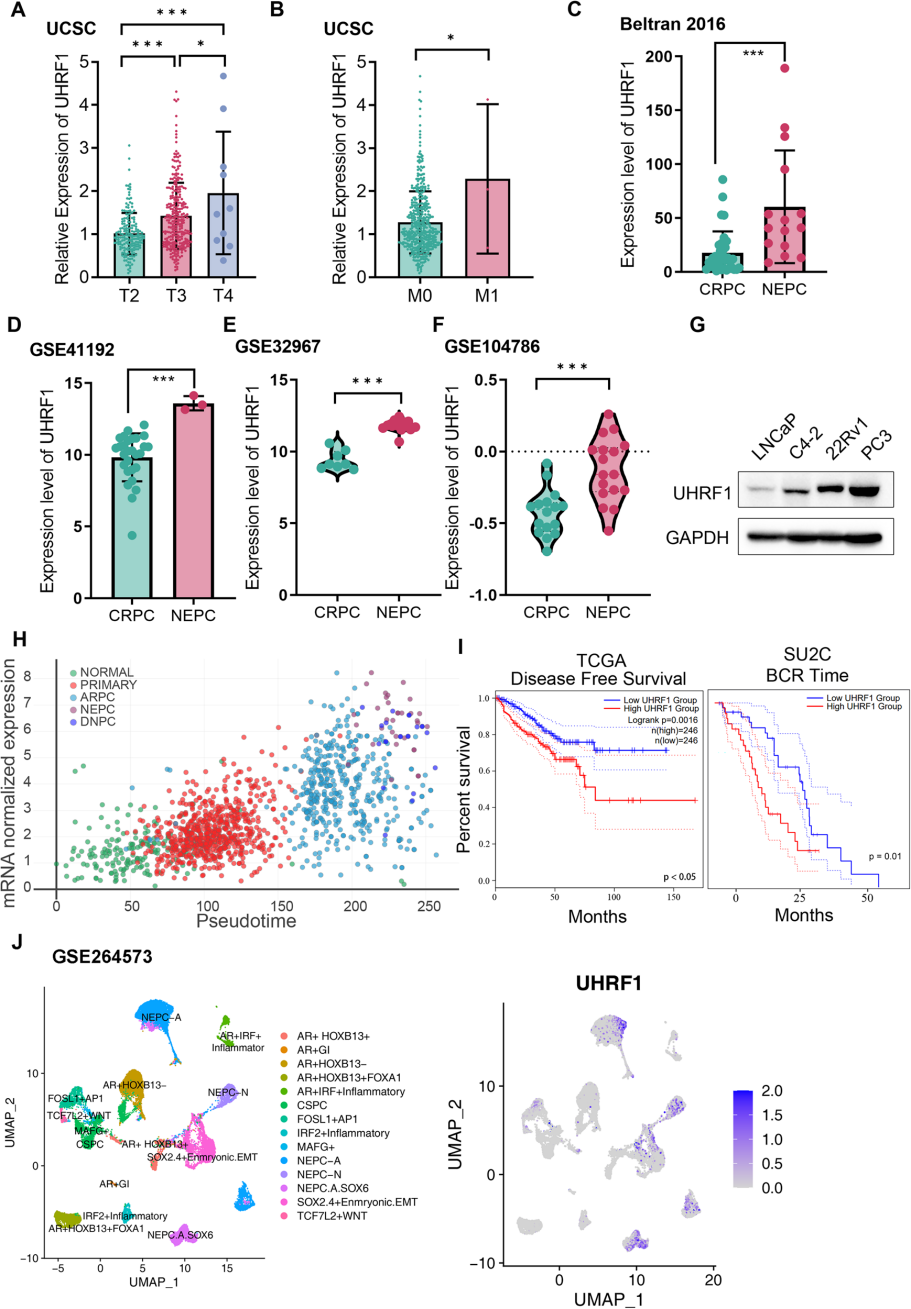
UHRF1 increases with the progression of prostate cancer. **A** Expression of UHRF1 in tumor tissues of prostate cancer patients at different stages. These data were derived from the TCGA database. **B** Expression of UHRF1 in tumor tissues of patients with prostate cancer with different metastatic states. These data were derived from TCGA database. **C**–**F** Expression of UHRF1 in tumor tissues of patients with prostate cancer at three different stages. These data were obtained from the GSE41192, GSE32967, GSE104786 datasets and cBioPortal. **G** Western Blot analysis of UHRF1 expression levels in prostate cancer cell lines LNCaP, C4-2, 22RV1, and PC3. **H** Pseudo-time series analysis of UHRF1 expression with prostate cancer progression. These data were derived from the Prostate Cancer Atlas (https://prostatecanceratlas.org/app/home). **I** (Left)Kaplan-Meier survival analysis of high and low expression of UHRF1 in prostate cancer (PRAD) patients from TCGA database (log-rank *p* = 0.0016). The data were obtained from GEPIA2 (http://gepia2.cancer-pku.cn). (Right) Kaplan–Meier analysis of biochemical recurrence (BCR)-free survival in SU2C prostate cancer patients stratified by UHRF1 expression. Patients with high UHRF1 expression showed significantly shorter BCR-free survival compared with those with low UHRF1 expression (log-rank *p* < 0.05). **J** (Left) UMAP projection of single cells from the prostate cancer dataset GSE264573, with cells colored and annotated by their respective cell states. (Right) Feature plot showing the normalized expression of UHRF1 onto the same UMAP layout. The color scale indicates the level of gene expression.

Based on TCGA database, we observed that elevated UHRF1 expression was associated with advanced stage or distant metastasis of PCa (Fig. 1A, B). Furthermore, we found in four public databases that UHRF1 expression levels in NEPC-staged PCa were significantly higher than those in the adenocarcinoma stage (Fig. 1C–F) [15]. We also conducted a pseudo-chronological analysis using a website (https://www.pcaprofiler.com) [16], and found that the expression of UHRF1 increased with PCa progression as well (Fig. 1H). The association between UHRF1 expression and patient prognosis was evaluated using disease-free survival (DFS) data from the TCGA cohort and biochemical recurrence (BCR)-free survival data from the SU2C cohort. Kaplan–Meier survival analysis revealed that high UHRF1 expression was significantly associated with shorter DFS and earlier BCR compared with low UHRF1 expression (Fig. 1I). We also examined the expression of UHRF1 in several PCa cell lines (Fig. 1G), and found low UHRF1 expression in LNCaP (a hormone-sensitive PCa) and higher expression in C4-2 and 22Rv1(two castration-resistant PCa) and the highest expression of UHRF1 in PC3 (AR-negative PCa cell line with partial NE features), suggesting that UHRF1 expression increased with PCa progression. To investigate the role of UHRF1 in PCa lineage plasticity, we analyzed a published single-cell RNA sequencing dataset (GSE264573). The results showed significant heterogeneity in UHRF1 expression across various cell subpopulations of PCa. Uniform Manifold Approximation and Projection (UMAP) analysis clearly revealed that UHRF1 expression is predominantly enriched in neuroendocrine prostate cancer (NEPC) cell clusters, particularly showing high expression in the ASCL1-driven NEPC-A and NEUROD1-driven NEPC-N subtypes (Fig. 1J). To further confirm the association between UHRF1 and the neuroendocrine lineage, we analyzed its co-expression with key neuroendocrine lineage-defining transcription factors. The results indicated that the expression hotspots of UHRF1 highly overlap with those of neuroendocrine and stemness markers, such as ASCL1, INSM1, SOX2, ONECUT2, and PEG10 (Figure S5B). Pearson correlation analysis of gene expression confirmed a positive correlation between the expression levels of UHRF1 and these master regulators (Figure S5C). Furthermore, an analysis of gene regulatory network activity across different cell subtypes showed that UHRF1 shares a highly consistent activity pattern with neuroendocrine gene modules driven by transcription factors like ASCL1 and SOX2 (Figure S5A).

### UHRF1 regulates the sensitivity of PCa to enzalutamide

**Fig. 2:**
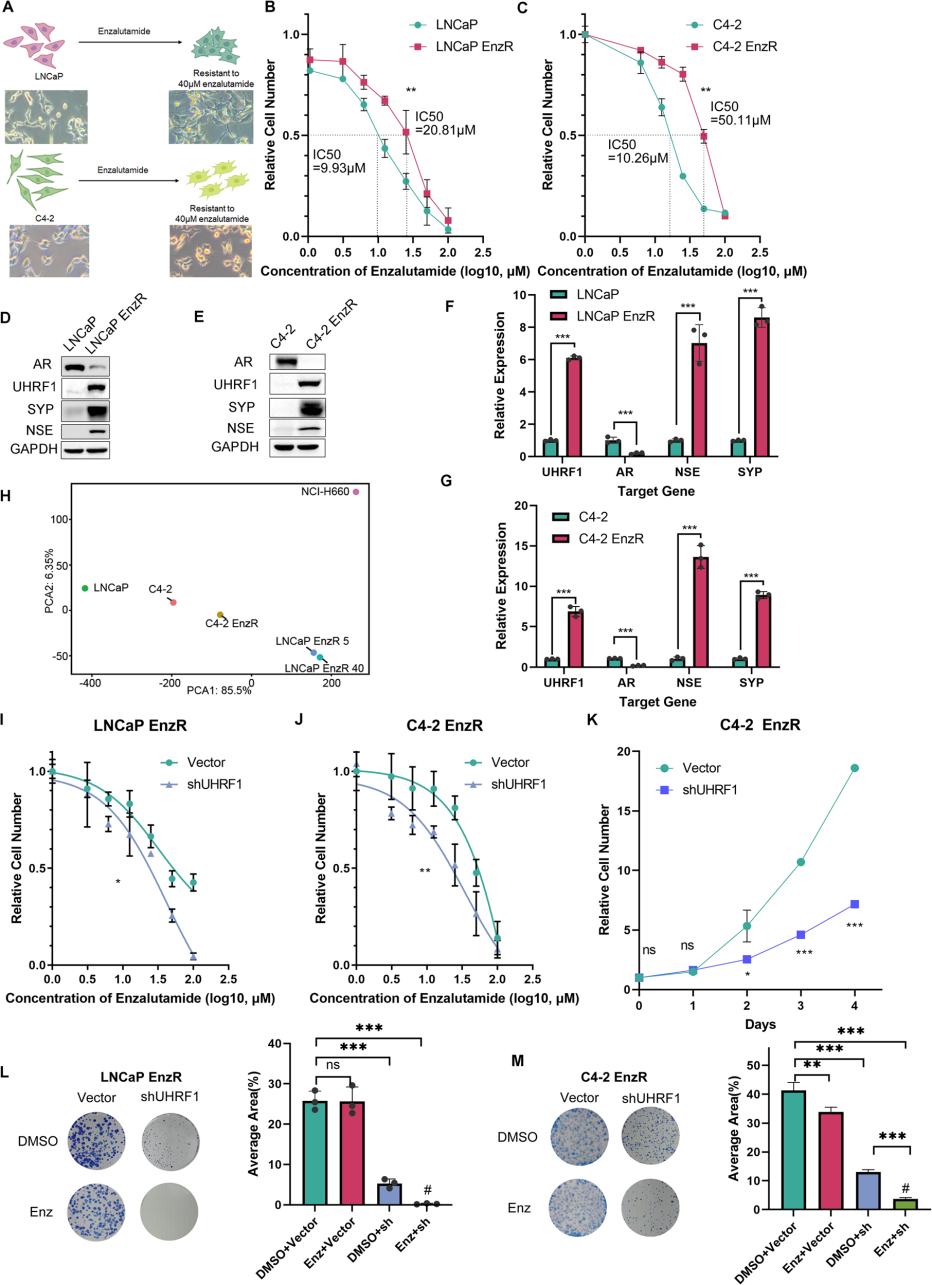
UHRF1 regulates the sensitivity of prostate cancer to enzalutamide. **A** Culture process and morphology of LNCaP-EnzR and C4-2-EnzR cell lines. **B** Enzalutamide IC50 determination of LNCaP and LNCaP-EnzR. **C** Enzalutamide IC50 determination for C4-2 and C4-2-EnzR. All cells were treated with increasing concentrations of enzalutamide for 7 days, then CCK8 was added for 2 h, and the absorbance at 450 nm was measured. **D**, **E** Western blot analysis of AR, UHRF1, SYP, and NSE expression levels in LNCaP and LNCaP-EnzR, C4-2 and C4-2-EnzR. **F**, **G** qPCR analysis of AR, UHRF1, SYP, and NSE expression levels in LNCaP, LNCaP-EnzR, C4-2, and C4-2-EnzR cells. **H** Principal component analysis of RNA-seq data from LNCaP, C4-2, their enzalutamide-resistant sublines, and NCI-H660 cells. **I**, **J** Effects of the negative control or UHRF1 knockdown on cell viability in LNCaP-EnzR and C4-2-EnzR. All cells were treated with increasing concentrations of enzalutamide for 7 days, then CCK8 was added for 2 h, and the absorbance at 450 nm was measured. **K** Effects of UHRF1 knockdown and enzalutamide on the viability of C4-2-EnzR cells. After transfection with vector or shUHRF1 plasmid, cells were re-inoculated, and cck8 was added to some cells every 24 h and incubated for 2 h, after which the absorbance at 450 nm was measured. **L**–**M** Colony formation analysis of UHRF1 knockdown and enzalutamide treatment in LNCaP-EnzR and C4-2-EnzR. After transfection with vector or shUHRF1 plasmid, cells were re-inoculated and treated with DMSO or enzalutamide for 14 days.

To in vitro recapitulate the enzalutamide resistance development and investigate the underlying molecular mechanisms, we have established two enzalutamide resistant cell lines LNCaP-EnzR and C4-2-EnzR by culturing LNCaP and C4-2 cells with increasing concentrations of enzalutamide for an extended period (Fig. 2A). The resulting resistant cells exhibited stable growth after 40 μM enzalutamide selection and both C4-2-EnzR and LNCaP-EnzR had higher IC50 values than parental control cells (Fig. 2B, C). Further analysis revealed that, as expected, the protein level of AR in both LNCaP-EnzR and C4-2-EnzR drastically decreased compared to the parental control cells, whereas UHRF1 and NE markers SYP and NSE were significantly increased at both protein and mRNA levels (Fig. 2D–G). RNA-seq and principal component analysis results revealed differences in transcriptional signatures between LNCaP, C4-2, LNCaP-EnzR, C4-2-EnzR cells, and the NEPC cell line NCI-H660. Both LNCaP-EnzR and C4-2-EnzR were far away from their parental cells. LNCaP-EnzR and C4-2-EnzR were not completely close to NCI-H660, but PCA1 explained 85.5% of the gene expression variation, and in this dimension both LNCaP-EnzR and C4-2-EnzR were closer to NCI-H660 than their parents. Therefore, LNCaP-EnzR and C4-2-EnzR have partial NE characteristics. Some differences between LNCaP-EnzR and C4-2-EnzR and NCI-H660 may reveal the heterogeneity of advanced PCa caused by the activating lineage plasticity gene program, and tumor cells show multiple characteristics such as NE or stem cells (Fig. 2H). We also used the R package BayesPrism to perform deconvolution analysis of bulk RNA-seq based on GSE264573 dataset by cell type and state (Figure S1A, B) [17], showed that both LNCaP-EnzR and C4-2-EnzR were mixtures of cells with multiple transcriptional signatures, including NEPC and stem-like AR-negative CRPC. These results suggest that long-term enzalutamide treatment leads to the switching of adenocarcinoma cells LNCaP and C4-2 into AR-negative PCa cells.

We next examined whether UHRF1 maintains drug resistance for LNCaP-EnzR and C4-2-EnzR cells. After knocking down UHRF1 in C4-2-EnzR and LNCaP-EnzR cells, cell viability was measured with increasing concentrations of enzalutamide treatment. Although both LNCaP-EnzR and C4-2-EnzR cells exhibited greater resistance to enzalutamide, they remained susceptible to enzalutamide after UHRF1 knockdown (Fig. 2I, J). UHRF1 knockdown also led to a slower proliferation of C4-2-EnzR and LNCaP-EnzR cells in responding to enzalutamide treatment (Fig. 2K, Figure S2G). Colony formation assay further demonstrated that while enzalutamide was only marginally effective in inhibiting the proliferation of LNCaP-EnzR and C4-2-EnzR cells, UHRF1 knockdown significantly restored the inhibitory effect of enzalutamide (Fig. 2L, M). Enzalutamide is able to induce apoptosis in PCa cells [18], however, enzalutamide treatment alone or in combination with UHRF1 knockdown did not substantially increase the apoptosis of LNCaP-EnzR and C4-2-EnzR (Figure S1C–H). The distribution across G0/G1 phases showed no significant differences between control and shUHRF1 cells, suggesting UHRF1 primarily affects AR signaling rather than proliferation (Figure S1I, J). Inhibitory effect of UHRF1 knockdown combined with enzalutamide on enzalutamide-resistant cells is achieved by inhibiting proliferation rather than increasing apoptosis. These findings suggest that UHRF1 plays a critical role in maintaining the drug resistance in the progression of PCa.

### UHRF1 promotes ubiquitination and degradation of AR

**Fig. 3:**
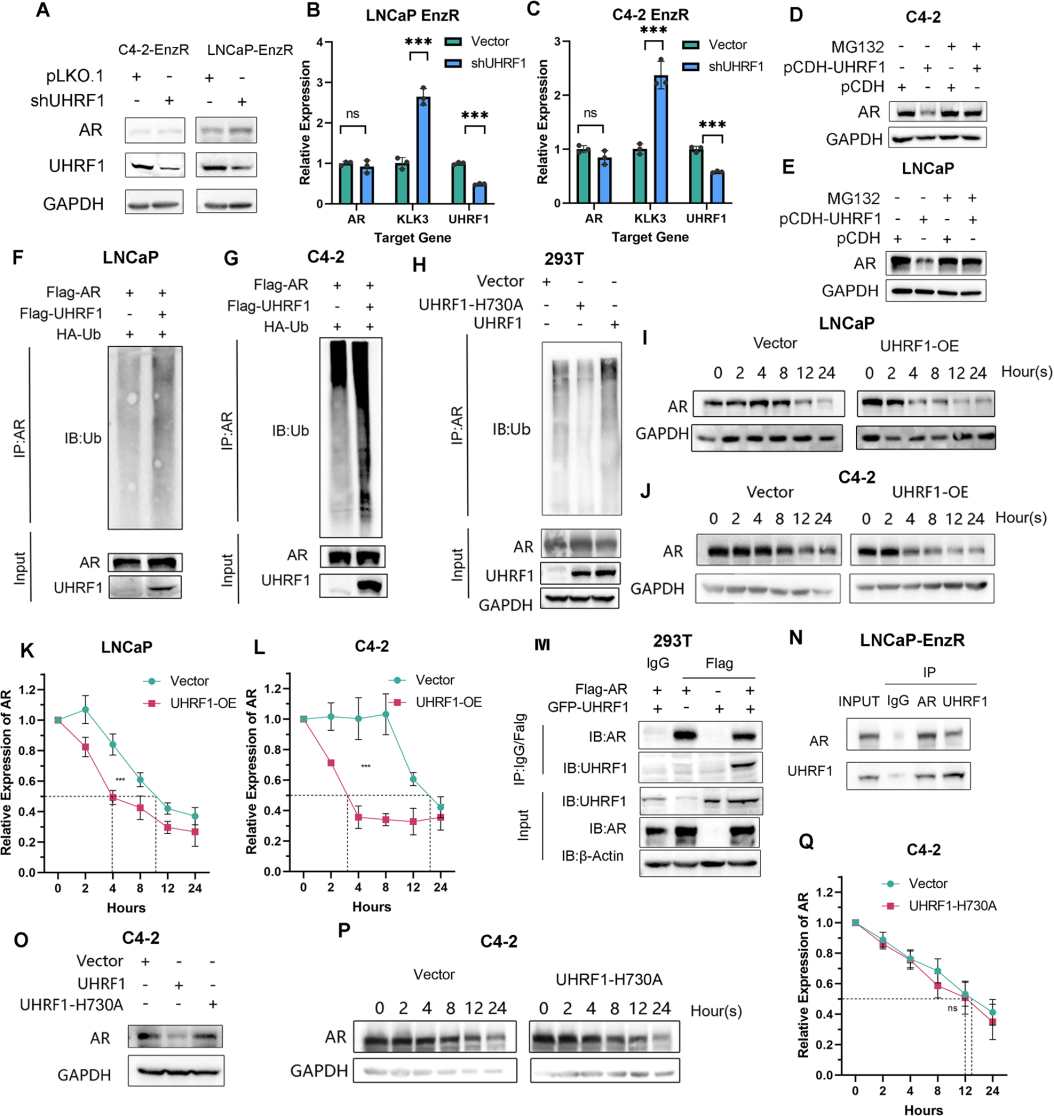
UHRF1 promotes AR ubiquitination and consequently AR degradation. **A** Western Blot analysis of AR and UHRF1 expression levels following UHRF1 knockdown in LNCaP-EnzR and C4-2-EnzR cells. **B**, **C** RT-qPCR results of AR, UHRF1, KLK3 mRNA levels following UHRF1 knockdown in LNCaP-EnzR and C4-2-EnzR. **D**, **E** Western blot analysis of AR expression levels following UHRF1 knockdown and MG132 treatment for 48 h in LNCaP-EnzR and C4-2-EnzR. **F**, **G** LNCaP and C4-2 cells were transfected with GFP-UHRF1, Flag-AR, and HA-Ub, and AR ubiquitination was examined using co-immunoprecipitation (Co-IP). **H** 293 T cells were transfected with UHRF1-H730A, Flag-AR, and HA-Ub, and ubiquitination of AR was examined via Co-IP. **I**–**L** LNCaP and C4-2 cells were transfected with the negative control or GFP-UHRF1 and subsequently treated with cycloheximide (CHX), and the decay of the AR protein over time was analyzed via Western blot. **M** 293 T cells were transfected with GFP-UHRF1 and/or Flag-AR, and the interaction between UHRF1 and AR was determined using Co-IP in 293 T cells. **N** The interaction between UHRF1 and AR was determined using Co-IP in LNCaP-EnzR cells. **O** Western Blot analysis of UHRF1 expression following transfection of negative control plasmid, GFP-UHRF1, or UHRF1-H730A in C4-2 cells. **P**–**Q** C4-2 cells were transfected with negative control or UHRF1-H730A and subsequently treated with CHX. Decay of the AR protein over time was analyzed by Western blot.

To investigate how UHRF1 affects the sensitivity of PCa cells to enzalutamide, we examined the expression level of AR and its downstream pathways. We found that UHRF1 knockdown in LNCaP-EnzR and C4-2-EnzR cells partially restored AR protein expression but had little effect on AR mRNA levels (Fig. 3A–C), suggesting that UHRF1 regulates AR at the protein level, rather than at the transcriptional level. Additionally, the expression of AR downstream gene KLK3 (PSA) was upregulated and the NE markers NSE and SYP did not display substantial changes (Fig. 3B, C, S2A, B). Overexpression of UHRF1 in the two parental cells, C4-2 and LNCaP, resulted in a decrease in AR protein levels, which could be reversed by treatment with the proteasome inhibitor MG132 (Fig. 3D, E), suggesting that UHRF1 modulates AR protein expression through ubiquitin-dependent proteasome degradation. We then assessed the effect of UHRF1 on AR ubiquitination and found that overexpression of UHRF1 enhanced AR ubiquitination and shortened the half-life of AR (Fig. 3F, G, I–L). Co-immunoprecipitation confirmed the binding between AR and UHRF1 in 293 T cells (Fig. 3M) and LNCaP-EnzR cells (Fig. 3N). A previous report showed that UHRF1 exerts its ubiquitination function through the RING domain, and the UHRF1-H730A mutation has been reported to disrupt the E3 ligase activity of the RING domain [19]. We mutated the histidine 730 of UHRF1 to alanine and found that the UHRF1-H730A mutant failed to increase AR ubiquitination (Fig. 3H), nor did it affect AR degradation (Fig. 3O–Q). Taken together, these findings suggest that UHRF1 facilitates AR ubiquitination and degradation via its RING domain.

NSC232003 is a highly potent and cell-permeable inhibitor of UHRF1 that binds to the 5mC binding pocket within the SRA domain of UHRF1 and modulates DNA methylation in a cellular context [20]. Notably, NSC232003 failed to inhibit the transcription of UHRF1 and several UHRF1-related genes (Figure S2C), nor did it affect the proliferation of drug-resistant cells (Figure S2D), suggesting that the role of UHRF1 in drug-resistant cells may be independent of DNA methylation.

### UHRF1 regulates the activity of AR signaling

**Fig. 4:**
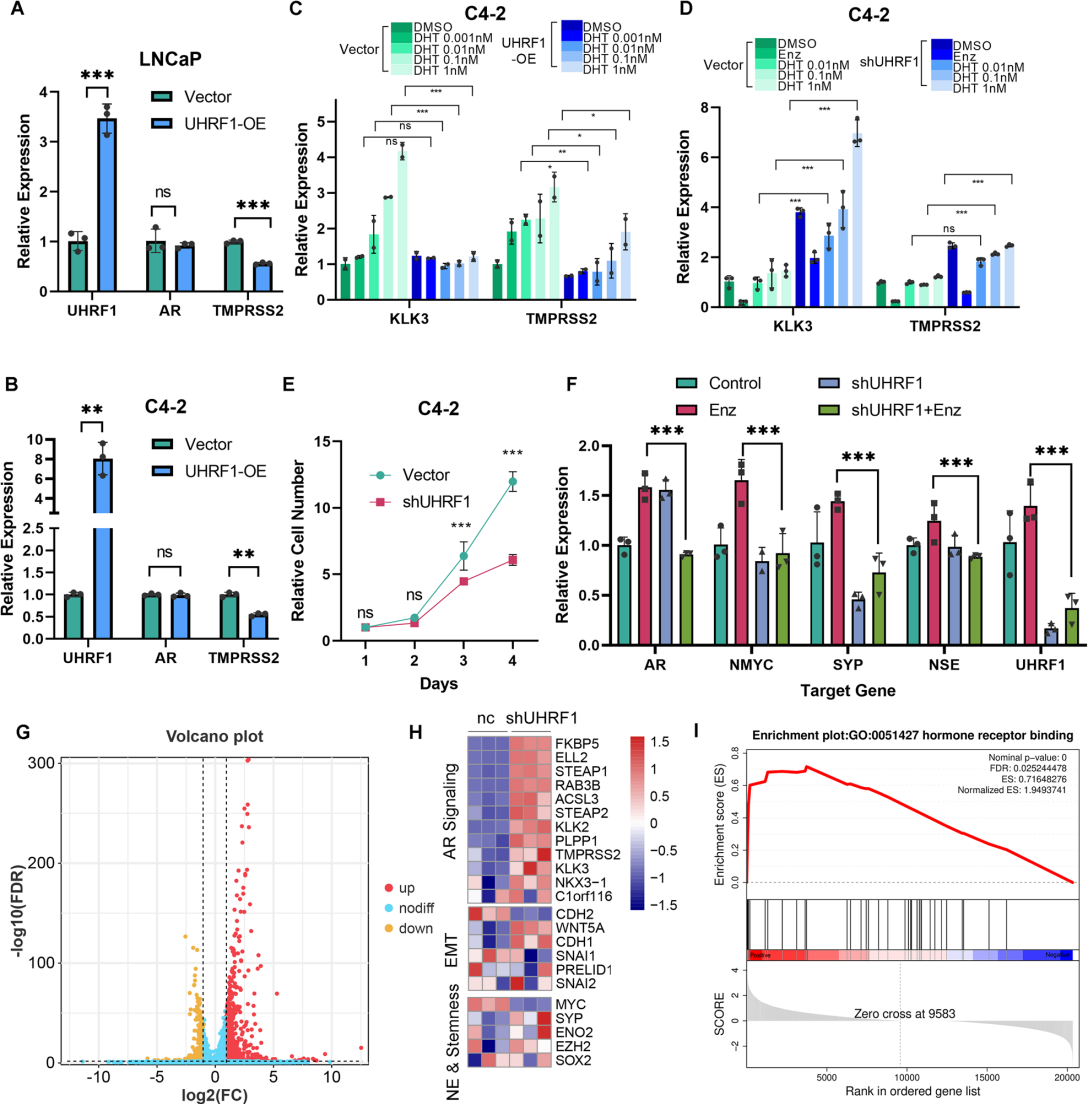
UHRF1 is essential for the development of enzalutamide resistance in prostate cancer. **A**, **B** RT-qPCR results of mRNA levels of various lineage markers in LNCaP and C4-2 cells 14 days after transfection with the negative control or UHRF1 overexpression plasmid. **C** Quantitative PCR detection of mRNA levels of AR downstream genes KLK3 and TMPRSS2 following treatment with varying concentrations of dihydrotestosterone after transfection with the negative control or UHRF1 overexpression plasmid in C4-2. **D** Quantitative PCR detection of the mRNA levels of AR downstream genes KLK3 and TMPRSS2 following treatment with varying concentrations of dihydrotestosterone after negative control or UHRF1 knockdown in C4-2 cells. **E** Cell viability assay of C4-2 cells following UHRF1 knockdown with 5 μM enzalutamide treatment. **F** mRNA expression levels of AR, NMYC, SYP, NSE, and UHRF1 in C4-2 cells after 14 days of treatment with 5 μM enzalutamide following UHRF1 knockdown. **G** Volcano plot of differentially expressed genes in NC and UHRF1 knockdown cells (*P* < 0.05, |log2FC | ≥ 1). **H** Heatmap of expression of some AR signaling, EMT, and NE & stem-like related genes. **I** GSEA results of hormone receptor binding gene set.

To investigate the role of UHRF1 in treatment-induced lineage switching of PCa, we found that UHRF1 overexpression resulted in partial attenuation of AR signaling and upregulation of specific NE and stemness-related factors in LNCaP and C4-2 cells (Fig. 4A, B, S2E, F). We subsequently treated UHRF1-overexpressing cells with increasing concentrations of dihydrotestosterone and found that the activation of AR downstream pathways, stimulated by increasing dihydrotestosterone concentrations, was inhibited by UHRF1 overexpression (Fig. 4C), while UHRF1 knockdown resulted in the opposite results (Fig. 4D). To further assess the functional role of UHRF1 in the development of enzalutamide resistance, we established stable C4-2 shUHRF1 cells and subsequently exposed them to increasing concentrations of enzalutamide. In contrast to the parental C4-2 cells, which rapidly exhibited adaptive responses to enzalutamide, UHRF1-depleted cells showed a delayed emergence of resistance, with slower proliferation recovery and attenuated induction of lineage plasticity and neuroendocrine markers (Fig. 4E, F). These findings suggest that UHRF1 facilitates early transcriptional reprogramming events that promote adaptation to AR pathway inhibition.

To explore the overall effect of UHRF1 on the transcriptome, we performed a RNA sequencing of control and UHRF1 knockdown cells. Compared to the control group, 825 genes were upregulated, and 277 genes were downregulated in UHRF1 knockdown cells (Fig. 4G and S3A). Further GO and KEGG analyses revealed that the downregulated genes were primarily involved in pathways related to DNA damage repair, including the cell cycle and DNA replication (Figure S3B–D). We selected a gene set from the literature to assess AR pathway activity, and the results indicated an increase in AR pathway activity following UHRF1 knockdown (Fig. 4H) [21]. UHRF1 knockdown has also been reported to suppress the expression of EMT-related genes CDH2 and SNAIL1, while activating WNT5A and CDH1. In terms of cell stemness and NE-related genes, UHRF1 knockdown led to the downregulation of MYC (Fig. 4H). Gene Set Enrichment Analysis (GSEA) revealed the upregulation of hormone receptor binding pathway and downregulation of cell cycle and DNA replication pathways (Fig. 4I, S3E, F). Additionally, GSEA showed an enrichment in MYC TARGETS and E2F TARGETS (Figure S3D). UHRF1 knockdown cells also exhibited a trend toward increased AR signaling, stem cell differentiation, and MET pathway activity, along with decreased stemness maintenance and EMT-related signatures. However, these changes did not reach strong statistical significance (Figure S6A–C). In PCa patient samples, UHRF1 expressions were also found to correlate with E2F family members such as E2F1 and E2F8 (Figure S3G). To further evaluate the relevance of UHRF1-regulated transcriptional programs in human PCa, we derived a UHRF1 gene signature from the differentially expressed genes identified in shUHRF1 RNA-seq data. When applied to the SU2C/PCF cohort, patients with high UHRF1 signature scores showed enrichment of neuroendocrine-related transcriptomic programs and tended to have poorer clinical outcomes (Figure S6D). These findings indicate that the transcriptional profile regulated by UHRF1 in resistant cell models is recapitulated in patient samples, supporting its association with lineage plasticity and aggressive disease behavior.

### Targeting UHRF1 improves the efficacy of enzalutamide in PCa

**Fig. 5:**
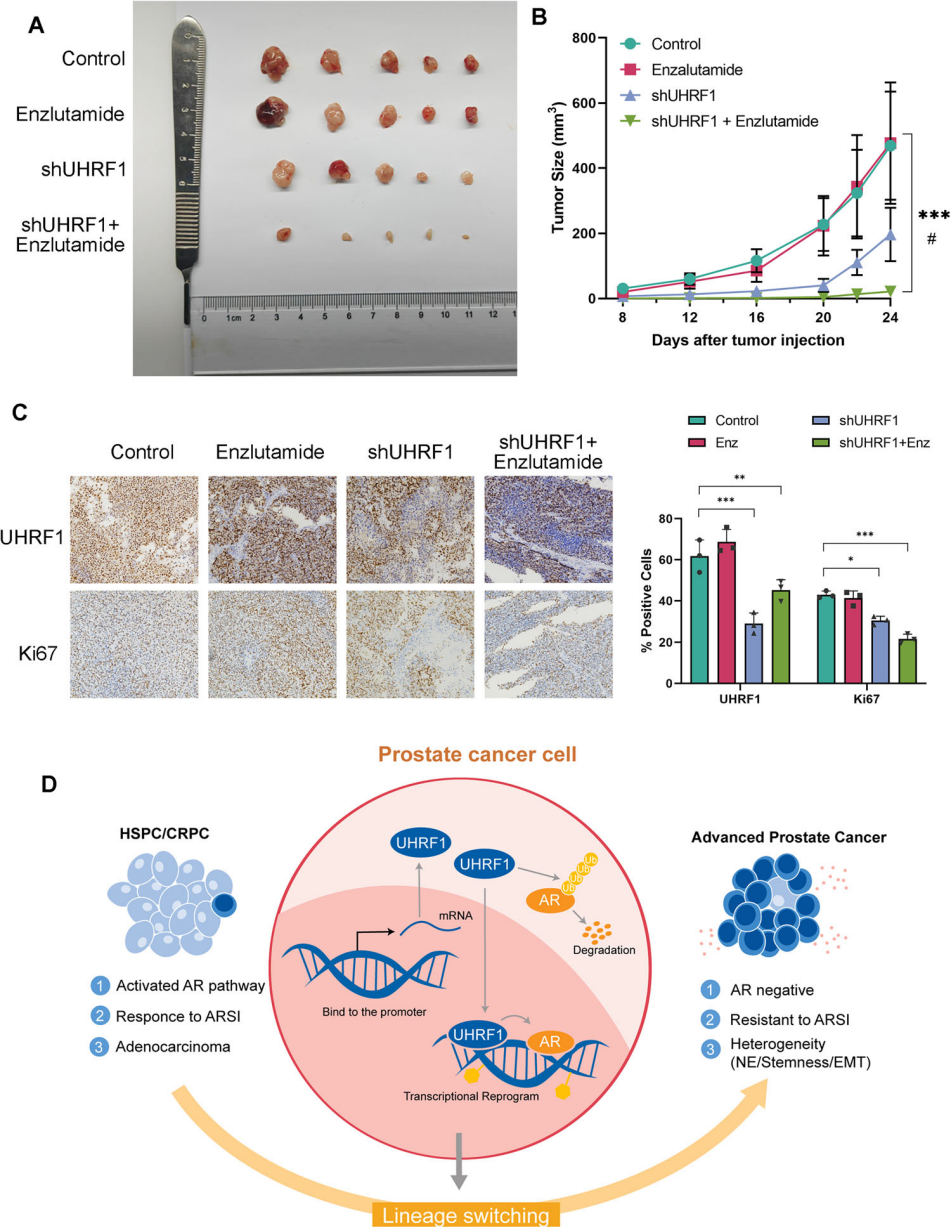
Inhibition of UHRF1 demonstrates potential therapeutic value in the treatment of advanced prostate cancer. **A** Representative images of tumors derived from NC- and UHRF-knockdown cells. **B** Growth curves of cell-derived xenografts (CDX) in tumors derived from NC and UHRF1 knockdown cells. **C** Immunohistochemical staining of UHRF1, and Ki67 in tumor tissues derived from NC and UHRF1 knockdown cells. **D** Proposed model: UHRF1 promotes the ubiquitination of AR, thereby facilitating its degradation and promoting cancer cell proliferation through AR-independent compensatory mechanisms, thus contributing to tumor treatment resistance.

To further investigate the role of UHRF1 in vivo, we assessed the sensitivity of the control and UHRF1 knockdown C4-2-EnzR xenografts in nude mice. Compared to the control group, UHRF1 knockdown combined with enzalutamide treatment significantly inhibited tumor growth. As shown in Fig. 5A, enzalutamide monotherapy had minimal inhibitory effects on C4-2-EnzR xenografts, confirming the resistant phenotype observed in vitro. In contrast, UHRF1 knockdown significantly reduced tumor growth, and the combination of UHRF1 knockdown and enzalutamide produced the most pronounced and sustained suppression of tumor progression, with a markedly lower tumor volume and weight at endpoint (Fig. 5B).

To gain deeper insights into tumor biology, we performed Ki67 immunohistochemistry to assess proliferation and Western blot analysis to evaluate neuroendocrine differentiation markers. Quantitative analysis of Ki67-positive nuclei demonstrated a significant decrease in proliferating cells in the UHRF1 knockdown plus enzalutamide group compared to all other groups (Fig. 5C). Western blotting confirmed that UHRF1 protein expression in xenograft tumors and was accompanied by increased ENO2, suggesting partial reversal of the neuroendocrine-like phenotype observed in resistant tumors (Figure S4C). These in vivo findings together with our transcriptomic and functional assays indicate that UHRF1 plays a pivotal role in maintaining the enzalutamide-resistant and neuroendocrine-like state of PCa, and its inhibition can re-sensitize resistant tumors to AR pathway blockade.

## Discussion

UHRF1 is primarily recognized for its crucial function of directing DNMT1 to DNA replication forks, thereby maintaining DNA methylation. It exhibits aberrant overexpression in PCa specimens compared to non-malignant prostate tissues. UHRF1 expression increases progressively with advancing malignancy and the development of drug resistance. UHRF1 functions as an oncogene that promotes PCa progression. Downregulation of UHRF1 diminishes the recruitment of DNMT1 to CpG islands, consequently reducing the maintenance of DNA methylation and restoring the expression of epigenetically silenced tumor suppressor genes such as RARB, CDH1, and PSP94 [22]. UHRF1 regulates CDC6 gene transcription as an AR coactivator and promotes anti-AR drug resistance through recruitment of the demethyltransferase KDM4C [23]. AKT1 promotes abiraterone resistance in PCa by regulating UHRF1 phosphorylation [24]. As DNA methylation is required for gene silence, the elevated expression of UHRF1 in proliferating and cancer cells strongly suggests that its role extends beyond that of DNA methylation [14]. Previous studies have mainly focused on the epigenetic function of UHRF1, such as DNA methylation maintenance and transcriptional repression via interaction with chromatin modifiers like EZH2 and LSD1. However, these findings primarily describe its role in regulating transcriptional programs rather than direct post-translational control of AR protein stability. Our results reveal that UHRF1 directly promotes AR polyubiquitination and proteasomal degradation through its RING domain, defining a novel post-translational mechanism distinct from previously described transcriptional regulation. Consequently, we investigated the potential association between UHRF1 and AR signaling pathway activity, and enzalutamide-induced PCa progression. While PCa typically develops through a gain in AR signaling after androgen deprivation therapy, a subset of CRPC will lose reliance on the AR, leading to AR-indifferent disease, showing more malignant phenotypes such as NE, stemness or EMT [15, 25].

Our study explored a mechanism of AR disappearance. Within the cell, AR is primarily degraded through the ubiquitin-proteasome pathway [26]. To date, no studies have explored the role of UHRF1 in the process of AR signal loss induced by therapy in PCa. UHRF1 is known to promote the ubiquitination of certain non-histone proteins [12]. Here, we demonstrated that UHRF1 may act as an E3 ubiquitin ligase, facilitating the degradation of AR. UHRF1 was elevated in tumor tissues of patients with NEPC and enzalutamide-resistant cells. Our investigation revealed that following UHRF1 knockdown, the AR pathway in the resistant cells was reactivated, responsiveness to androgen stimulation was restored, and could be re-inhibited by enzalutamide. Therefore, the increased UHRF1 promotes the loss of AR and facilitates the differentiation of cells towards an AR-independent proliferative pathway.

Though UHRF1 knockdown did not significantly reduce the classical NE markers, we observed that knocking down UHRF1 abrogated the enzalutamide-induced upregulation of NE markers in C4-2 cells. Advanced PCa is highly heterogeneous and is a difficult problem for clinical treatment [27]. UHRF1 mediates the disappearance of AR signaling during PCa progression, regardless of whether these cells will subsequently become NE, stem-like cells or other. UHRF1 knockdown sensitized both C4-2 and C4-2-EnzR cells to enzalutamide, enhancing the drug’s inhibitory effect. Although UHRF1 may not be a direct driver of NE transformation, it plays a crucial role in the process of lineage switching. UHRF1 is important for the inactivation of the classical AR pathway and the maintenance of drug resistance. Inhibiting UHRF1 may prolong the effectiveness of enzalutamide and re-sensitize drug-resistant cells, potentially extending the duration of first-line treatment in clinical settings. Our study focuses on the transitional stage of treatment-induced lineage plasticity, before the complete neuroendocrine differentiation observed in terminal NEPC. Fu et al. (2023) investigated fully established NEPC, where UHRF1 expression is maintained by ASCL1 to stabilize the neuroendocrine phenotype [24]. Thus, our work complements and extends previous findings by uncovering the early mechanism through which UHRF1 initiates AR pathway loss and facilitates lineage switching during enzalutamide resistance.

Despite these findings, our study has limitations. Although UHRF1 suppression delayed the induction of lineage plasticity programs and enhanced enzalutamide sensitivity, it did not fully reverse the neuroendocrine phenotype in established enzalutamide-resistant cells. Our results therefore highlight that UHRF1 inhibition is most effective within the transitional window during which AR signaling is declining but not yet fully extinguished. In this intermediate state, lineage plasticity remains dynamic and therapeutically modifiable. Targeting UHRF1 during this window may slow or prevent irreversible progression to an AR-independent phenotype, thereby extending the therapeutic lifespan of AR pathway inhibitors. Thus, while UHRF1 inhibition alone cannot remodel fully established NEPC, it holds significant potential as a strategy to intercept lineage plasticity before terminal differentiation occurs.

In conclusion, our study has demonstrated that during the lineage switching of PCa induced by enzalutamide treatment, UHRF1, functioning as an E3 ubiquitin ligase, promotes AR ubiquitination degradation and inhibits AR pathway activity, thereby causing PCa to transition to an AR-independent lineage for proliferation (Fig.5D). Our study elucidated the role of UHRF1 in the remodeling of PCa lineages, suggesting the therapeutic potential of inhibiting UHRF1 at this critical juncture before AR completely disappears to reverse PCa lineage switching, and providing novel insights into treatment strategies for advanced PCa.

## Materials and methods

### Cell lines and cell culture

LNCaP, C4-2, CWR22Rv1, and HEK293T cells were purchased from Procell Life Science & Technology Co. Ltd. (Wuhan, China). All the cell lines were authenticated by STR profiling and tested negative for mycoplasma contamination. LNCaP, C4- 2, and CWR22Rv1 cells were cultured in RPMI-1640 medium supplemented with 10% fetal bovine serum (ExCellBio, Shanghai, China). HEK293T cells were cultured in DMEM supplemented with 10% fetal bovine serum. Enzalutamide and MG132 were purchased from MedChemExpress. Cycloheximide was purchased from Dalian Meilun Biotechnology Co. Ltd.

### Generation of LNCaP-EnzR and C4-2-EnzR cells

LNCaP or C4-2 cells were initially cultured in media containing 5 µM enzalutamide, and the drug concentration in living cells was increased by 5 μM after every 10 passages until the drug concentration reached 40 μM and passaged 10 times. The cells were named LNCaP-EnzR and C4-2-EnzR.

### Plasmids and primers

pcDH-UHRF1 was used to construct pcDH-UHRF1-H730A according to the instructions of the Q5® Site-Directed Mutagenesis Kit. The primer sequences used for the PCR are listed in Supplementary Table 1.

### Cell viability assay

Cell proliferation was measured using the CCK-8 assay following the manufacturer’s instructions. Cells (1 × 10³/well) were seeded in 100 μL medium in 96-well plates. After 12 h, the medium was replaced with varying concentrations of DMSO or enzalutamide and incubated for 24–72 h. Then, 10 μL of CCK-8 was added per well, and cells were incubated at 37 °C for 1–4 h. Absorbance at 450 nm was measured using an microplate reader (Tecan).

### Colony formation assay

LNCaP and C4-2 cells were seeded in six-well plates at a density of 1 × 10^3^ cells/well with different concentrations of DMSO or enzalutamide. After 14 days, the cells were washed twice with phosphate-buffered saline (PBS), fixed with 4% paraformaldehyde, and stained with 0.1% crystal violet.

### Co-Immunoprecipitation (co-IP)

For IP, cell lysates were prepared with agarose protein A  +  G Santa Cruz Biotechnology, Inc., Dallas, TX, USA), incubated with the primary antibody overnight at 4 °C, and mixed with agarose protein A  +  G for 6 h at 4 °C. After washing three times with lysis buffer, the loading buffer was added, and agarose protein A  +  G was removed via centrifugation.

### Western blot

Cellular proteins were extracted and separated by SDS-PAGE, then transferred to nitrocellulose membranes. After blocking with 5% milk in PBS containing 0.1% Tween-20 for 1 h at room temperature, membranes were incubated overnight at 4 °C with primary antibodies: AR (Santa Cruz Biotechnology Inc., Dallas, TX, USA; Cat# sc-7305, 1:1000; Cell Signaling Technology, Danvers, MA, USA; Cat# 5153S, 1:1000), UHRF1 (Proteintech Group, Rosemont, IL, USA; Cat# 21402-1-AP, 1:1000), Ub (ZenBio Inc., Durham, NC, USA; Cat# 382766, 1:1000), NSE (Proteintech Group; Cat# 10149-1-AP, 1:1000), SYP (Proteintech Group; Cat# 17785-1-AP, 1:1000), Flag (Vazyme Biotech Co., Ltd., Nanjing, Jiangsu, China; Cat# RA1003-01, 1:1000), and GAPDH (Santa Cruz Biotechnology Inc.; Cat# sc-47724, 1:1000) as a loading control. After incubation with appropriate secondary antibodies, immunoreactive bands were visualized using an enhanced chemiluminescence detection system.

### RNA extraction and qRT-PCR

Total RNA was extracted with TRIzol reagent (Invitrogen, USA), and 1 µg RNA was reverse transcribed using HiScript® III RT SuperMix (Vazyme Biotech Co., Ltd., Jiangsu, China). qPCR was performed with 2×SYBR Green Master Mix (Vazyme Biotech Co., Ltd., Jiangsu, China), and GAPDH was used for normalization. Statistical analysis was done using Student’s t-test, with *p*  <  0.05 considered significant. Primer sequences are listed in Supplementary Table 1.

### RNA sequencing

RNA samples were collected from NC and shUHRF1 LNCaP-ENzR cells. RNA isolation, library construction, and RNA sequencing (RNA-seq) were carried out following standard protocols. The library products were sequenced using a BGISEQ-500. Standard bioinformatics analysis was performed by Guangzhou Jidiao Biotechnology Co.; Differentially expressed genes were identified using DESeq2, with adjusted *p*-value < 0.05 and |log₂(fold change)| > 1 as the cutoff. Heatmaps were generated using the pheatmap package in R. KEGG pathway enrichment and Gene Set Enrichment Analysis (GSEA) were performed using the clusterProfiler package. KEGG terms and gene sets were considered significant at *p* < 0.05. BayesPrism R package was used to perform cell state deconvolution of bulk RNA-seq data from the GSE264573 dataset.

### Single-cell gene regulatory network and co-expression analysis

A publicly available single-cell RNA sequencing (scRNA-seq) dataset of castration-resistant prostate cancer (GSE264573) was downloaded from the Gene Expression Omnibus (GEO). The analysis was performed using the Seurat R package.

Gene expression data were log-normalized and scaled. Cell populations were visualized using Uniform Manifold Approximation and Projection (UMAP) based on the original study’s annotations. The expression of UHRF1 and key neuroendocrine markers was visualized on UMAP plots. The relationship between UHRF1 and these markers was assessed by calculating Pearson correlation coefficients from the expression data, which were then displayed as a heatmap. The association between UHRF1 expression and the activity of predefined gene modules across different cell clusters was similarly evaluated and visualized.

### Survival analysis

Kaplan-Meier survival analysis of high and low expression of UHRF1 in prostate cancer patients from TCGA database were obtained from GEPIA2 (http://gepia2.cancer-pku.cn). Gene expression and clinical data for the SU2C/PCF prostate cancer cohort were obtained from the cBioPortal database. Patients were stratified into high and low UHRF1 expression groups based on the median mRNA expression value. Biochemical recurrence (BCR)-free survival was analyzed using the Kaplan–Meier method and compared by log-rank test with the R package survival (v3.5) and survminer (v0.4.9).

### Generation and application of the UHRF1 signature

A UHRF1-regulated gene signature was generated from bulk RNA-seq data of control and shUHRF1-treated C4-2-EnzR cells. Genes with an absolute log₂ fold change ≥ 1 and adjusted *P* < 0.05 were defined as UHRF1-regulated genes (listed in Supplementary Table S3).

For validation, normalized transcriptome data from the SU2C/PCF prostate cancer cohort were analyzed. A UHRF1 signature score was calculated for each sample as the mean expression of upregulated genes minus that of downregulated genes. Patients were divided into high and low groups by the median score, and associations with neuroendocrine features and clinical outcomes were evaluated.

### Mouse models

20 four-week-old male nude mice (GemPharmatech, Nanjing, China) were housed under pathogen-free conditions with free access to food and water. All procedures were approved by Nanfang Hospital. For subcutaneous xenografts, 1 × 10⁶ C4-2-EnzR nc or UHRF1-knockdown cells mixed with Matrigel (1:1) were injected into the flanks. Mice were castrated post-injection and divided into control and enzalutamide-treated groups. Enzalutamide was administered intraperitoneally. Tumor size was measured every two days, and treatment continued until tumors reached 2000 mm³ or 1.5 cm in diameter. Mice were euthanized by CO₂ inhalation, and tumors were excised, measured, and analyzed to assess therapeutic effects. Tumor growth curves were generated from collected data.

### Immunohistochemistry

A total of 5-micron thick formalin-fixed paraffin-embedded human tissue sections were stained with AR (Santa Cruz Biotechnology Inc., catalog sc-7305), Ki67 (Servicebio, GB111499), UHRF1 (Proteintech, catalog 21402-1-AP), and antibodies per manufacturer’s instructions. The stained slides were digitized using a panoramic slice scanner (3DHISTECH, Hungary) with a 40× objective lens. The positive cell rate was calculated using an IHC profiler in ImageJ.

### Flow cytometry analysis of apoptosis

PCa cells were transfected with plasmids or treated with enzalutamide for 48 h. Cell apoptosis was analyzed with FITC Annexin V Apoptosis Detection Kit (KeyGEN BioTECH, Jiangsu, China). The stained cells were acquired by flow cytometry and analyzed by FlowJo software. The FITC Annexin V positive and PI negative or FITC Annexin V and PI positive were measured as apoptosis cells.

### Statistical analysis

All data are presented as the mean ± standard deviation. Statistical analyses were performed using Microsoft Excel (Microsoft Corp.). Differences between individual groups were analyzed using one-way analysis of variance (ANOVA). Statistical significance was set at *P*  <  0.05. Synergistic effects were determined using two-way analysis of variance (ANOVA) and the Bliss independence model. For all statistical analyses, differences are labeled as ns, not significant; *P < 0.05; **P < 0.01; ***P < 0.001. Statistical significance was set at *P* < 0.05. #Synergy by bliss-independent analysis.

## Supplementary information

Supplementary Figure 1

Supplementary Figure 2

Supplementary Figure 3

Supplementary Figure 4

Supplementary Figure 5

Supplementary Figure 6

Supplementary Figure Legends

Supplementary Table 1

Supplementary Table 2

Supplementary Table 3

Raw Data of Western Blot

## Data Availability

RNA-seq data are deposited in GEO (GEO accession number: GSE295751). Scripts and codes are available upon request.
