# Supplementary figures and images for: UHRF1 regulates AR ubiquitination to promote the loss of AR signaling and enzalutamide resistance in progression of prostate cancer
Source: Cell Death Dis. 2026 Feb 27;17(1):286. doi: 10.1038/s41419-026-08511-9 (PMC13031396; doi:10.1038/s41419-026-08511-9)

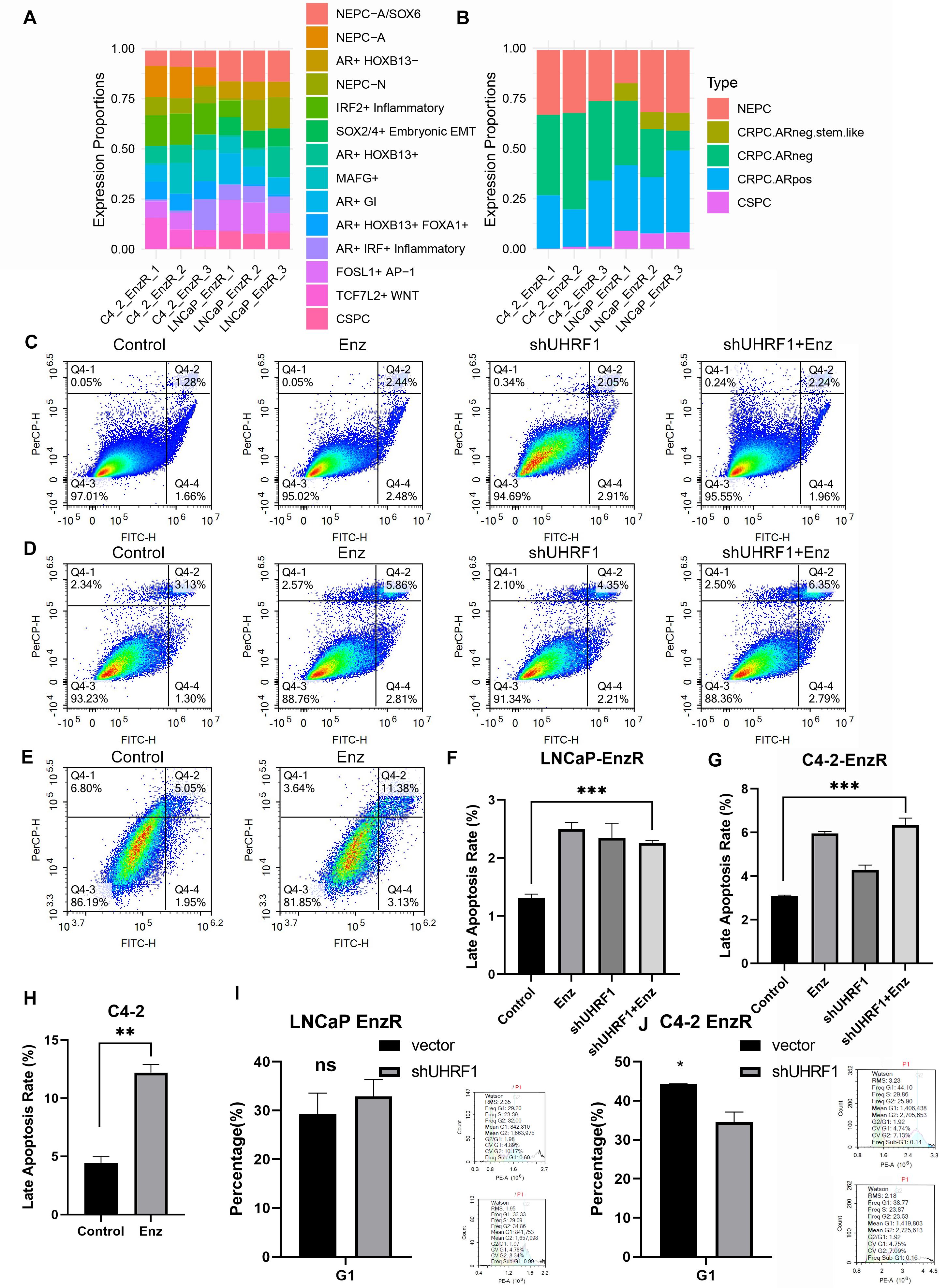

Supplement: Supplementary file 1 — Supplementary Figure 1 [file 41419_2026_8511_MOESM1_ESM.tif]

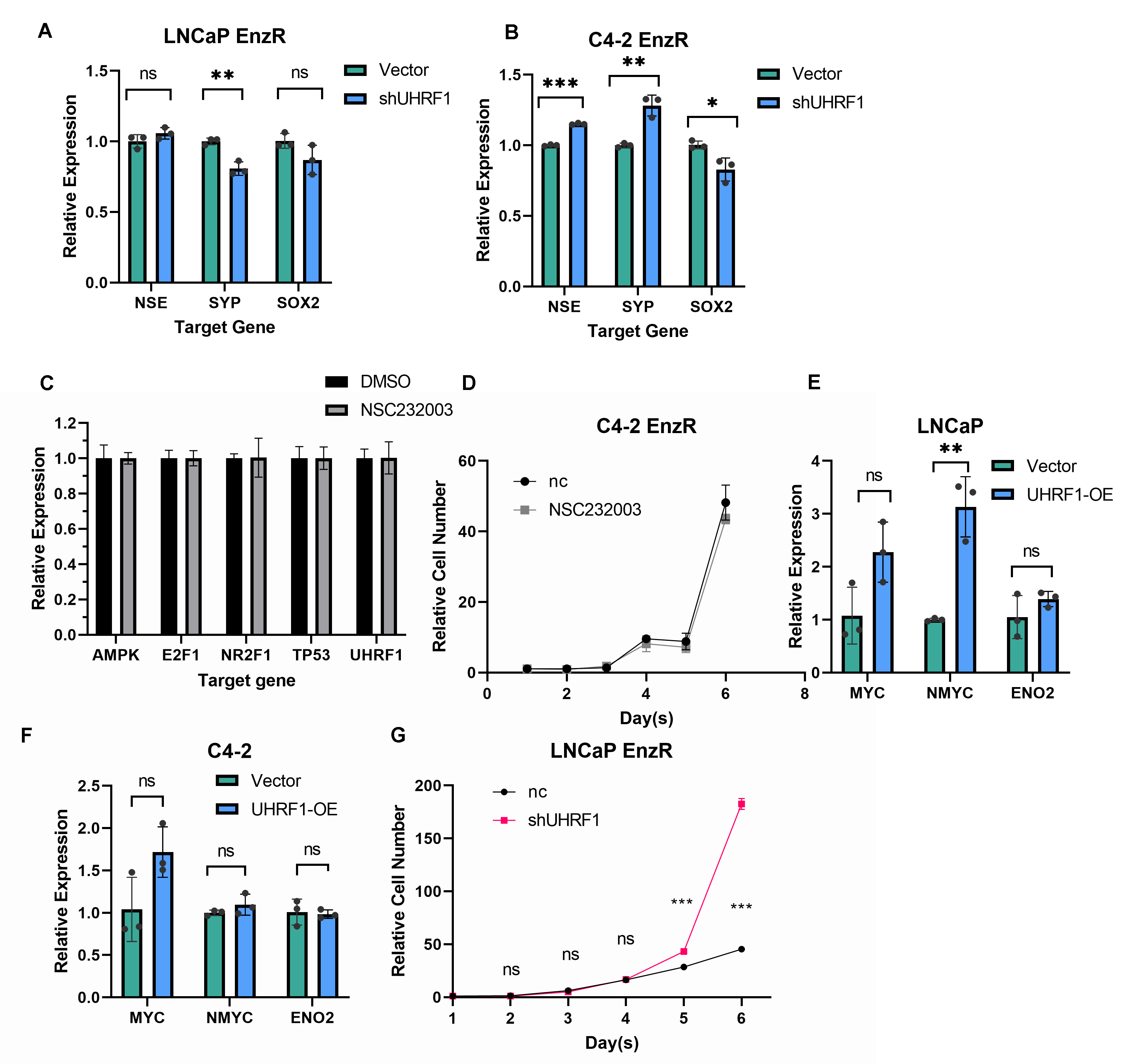

Supplement: Supplementary file 2 — Supplementary Figure 2 [file 41419_2026_8511_MOESM2_ESM.tif]

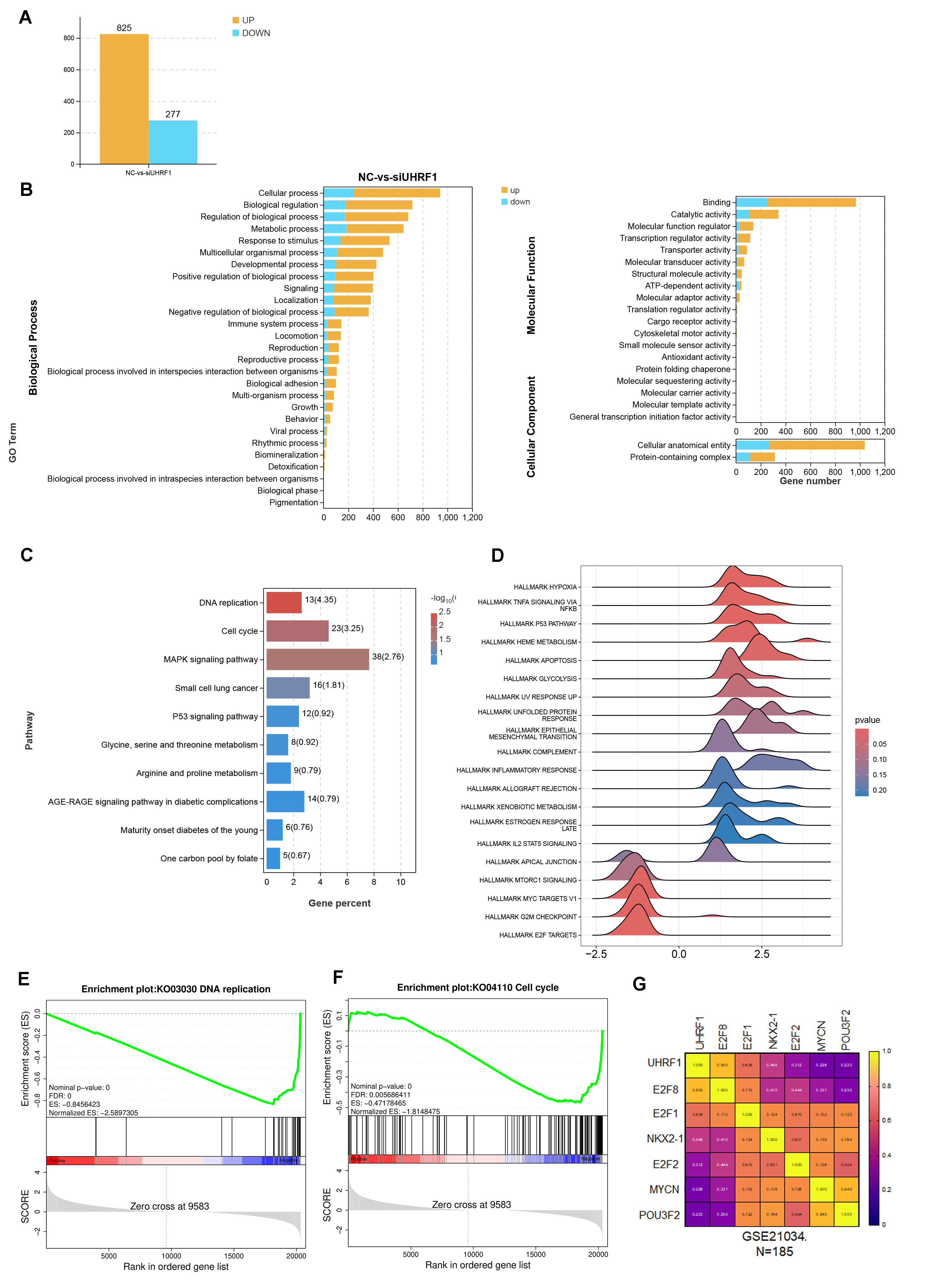

Supplement: Supplementary file 3 — Supplementary Figure 3 [file 41419_2026_8511_MOESM3_ESM.tif]

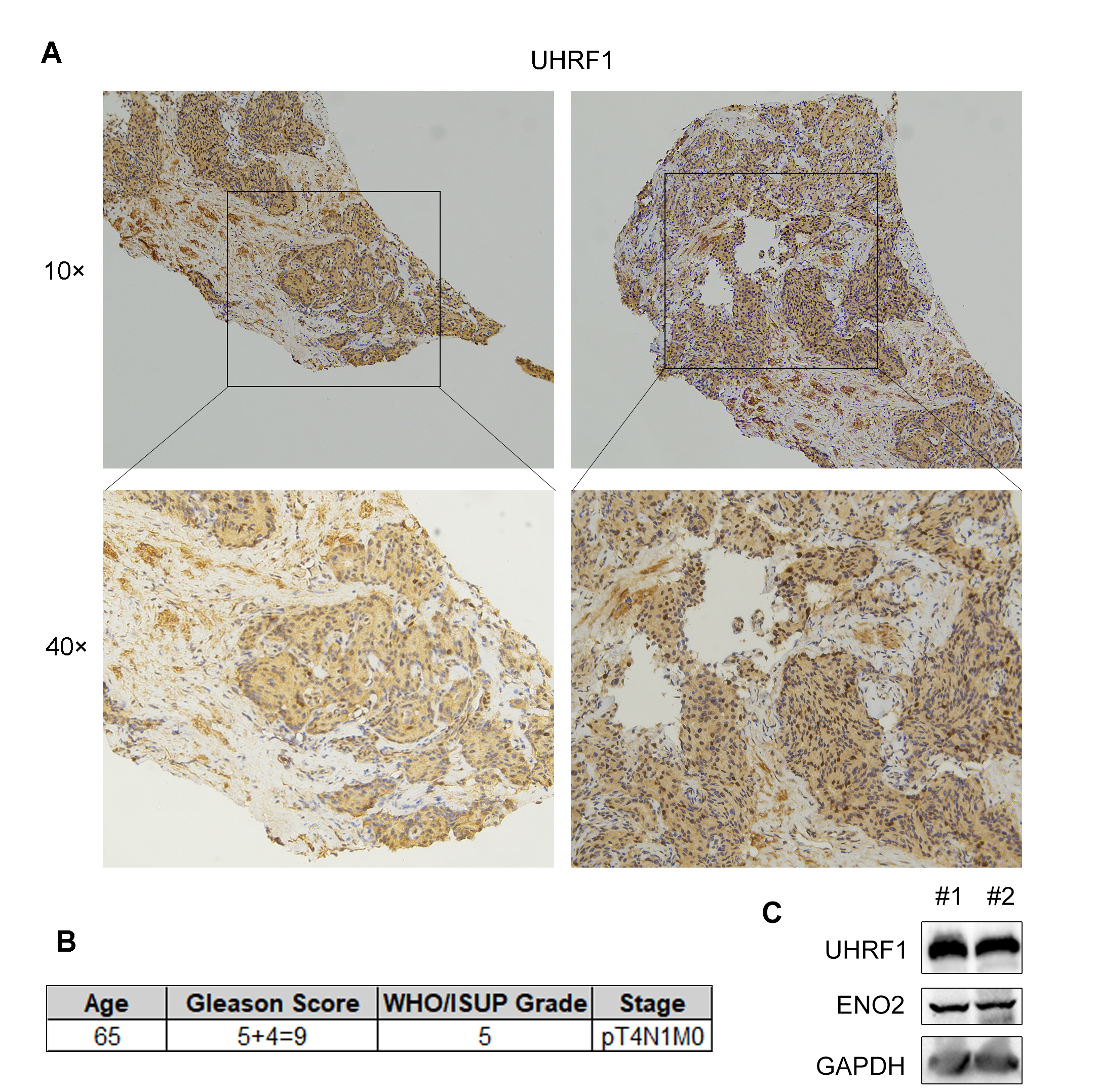

Supplement: Supplementary file 4 — Supplementary Figure 4 [file 41419_2026_8511_MOESM4_ESM.tif]

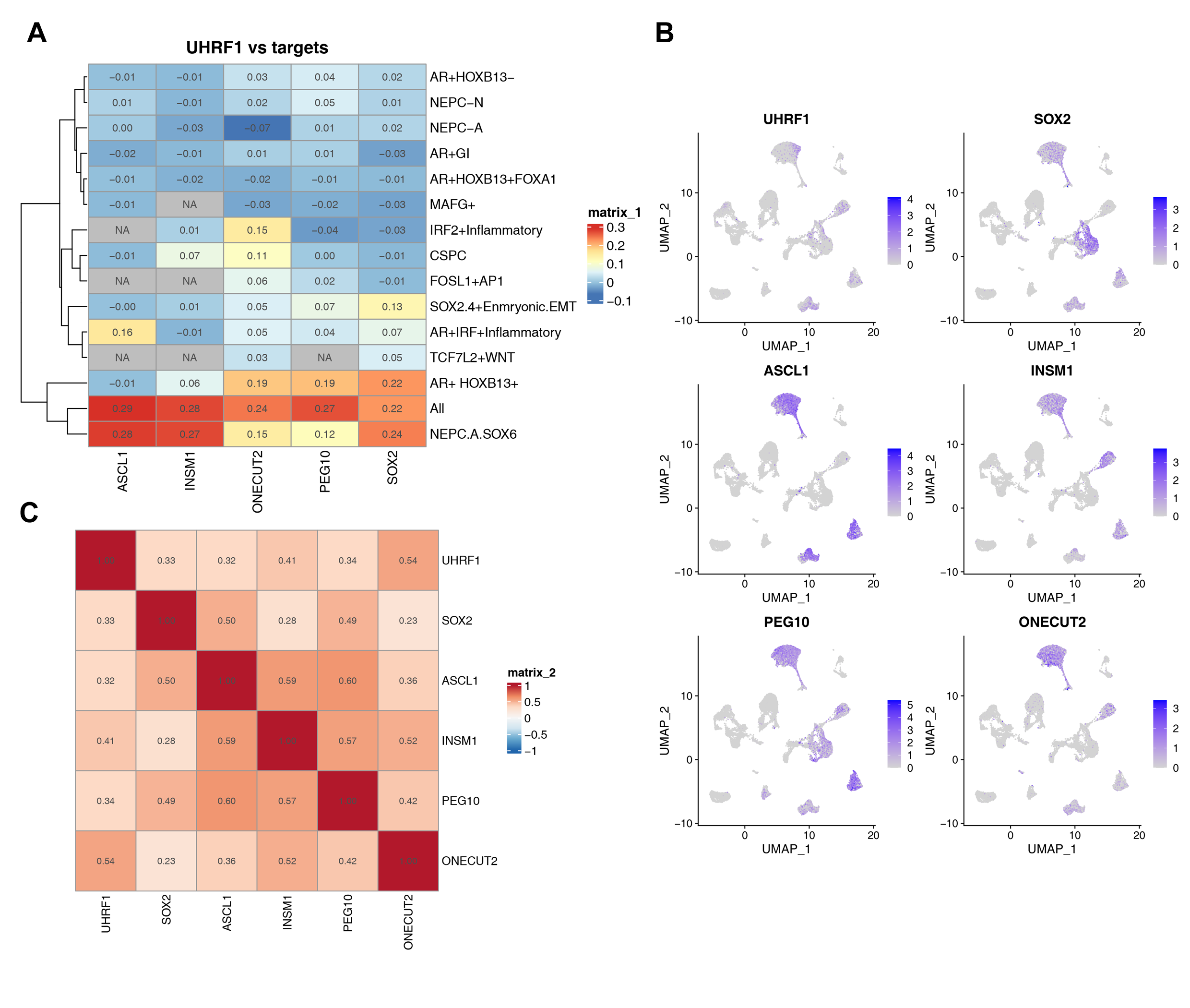

Supplement: Supplementary file 5 — Supplementary Figure 5 [file 41419_2026_8511_MOESM5_ESM.tif]

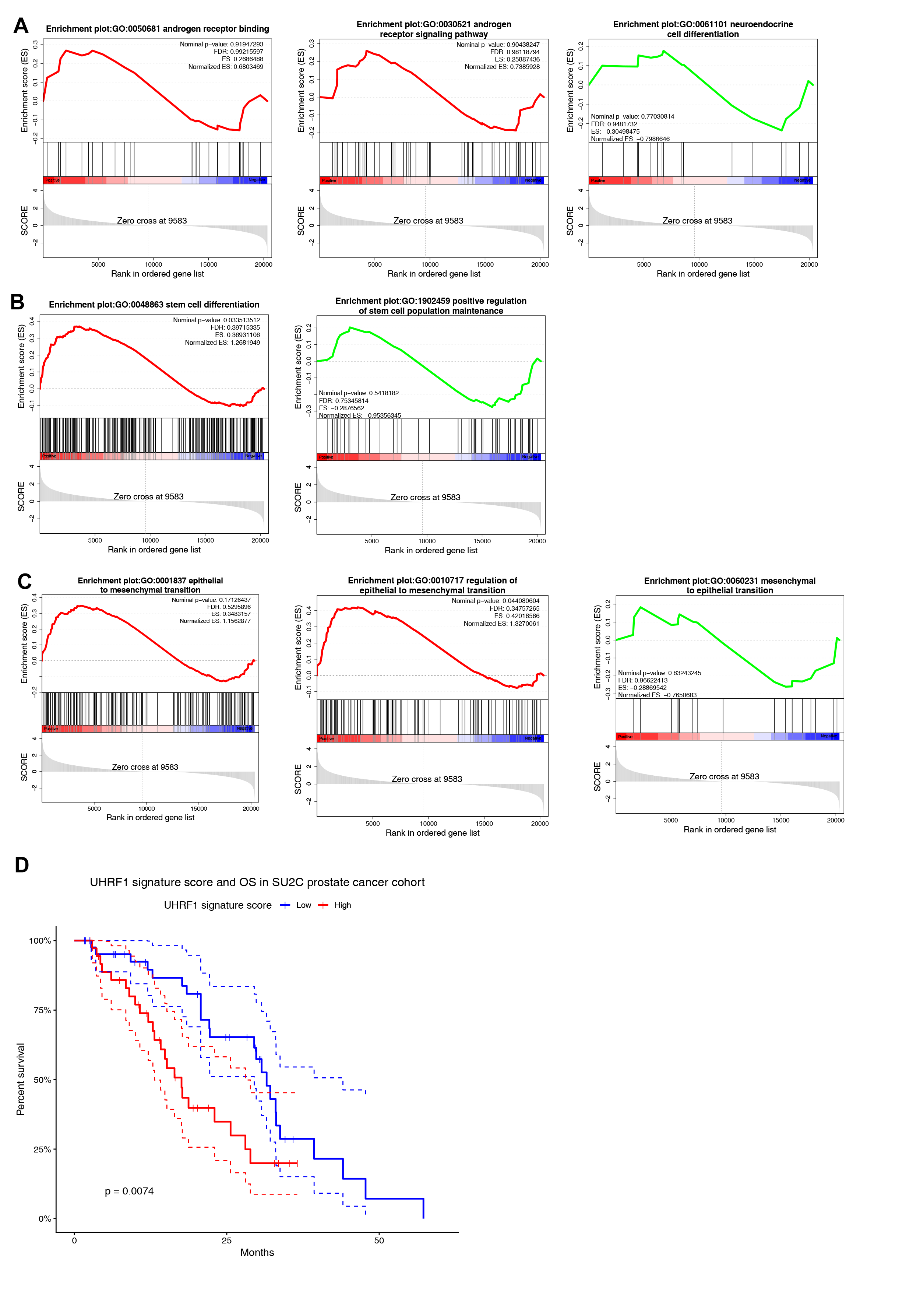

Supplement: Supplementary file 6 — Supplementary Figure 6 [file 41419_2026_8511_MOESM6_ESM.tif]
