# Supplementary material for: UHRF1 regulates AR ubiquitination to promote the loss of AR signaling and enzalutamide resistance in progression of prostate cancer
Source: Cell Death Dis. 2026 Feb 27;17(1):286. doi: 10.1038/s41419-026-08511-9 (PMC13031396; doi:10.1038/s41419-026-08511-9)
Supplement: Supplementary file 7 — Supplementary Figure Legends [file 41419_2026_8511_MOESM7_ESM.pdf]

Supplementary Figures

Figure S1 Heterogeneous cell state composition in enzalutamide-resistant prostate cancer and limited pro-apoptotic effect of UHRF1 knockdown

A) Expression proportions of 14 transcriptional programs (cell states) identified from the GSE264573 dataset using BayesPrism. Each bar represents a biological replicate from C4-2_EnzR or LNCaP_EnzR samples. B) Expression proportions grouped into five broader cell types: CSPC (Castration-Sensitive Prostate Cancer), CRPC.ARpos (Castration-Resistant Prostate Cancer with AR-positive signature), CRPC.ARneg (AR-negative CRPC), CRPC.ARneg.stem.like (stem-like AR-negative CRPC), and NEPC (Neuroendocrine Prostate Cancer). These groupings reflect lineage plasticity and resistance mechanisms across replicates. C-D) LNCaP-EnzR and C4-2-EnzR cells were transfected with vector or shUHRF1 plasmid, then treated with 10μM enzalutamide for 48 hours. The cells were then collected and incubated with PI and Annexin V-fluorescein isothiocyanate, and apoptosis was detected by flow cytometry. E) C4-2 cells were treated with DMSO or 10μM enzalutamide for 72 hours, then collected and incubated with PI and Annexin V-fluorescein isothiocyanate, and apoptosis was detected by flow cytometry. F-H) Corresponding statistical results of LNCaP-EnzR, C4-2-EnzR and C4-2 flow cytometry apoptosis analysis. I-J) Flow cytometry analysis of cell cycle distribution in control and shUHRF1 LNCaP-EnzR (I) and C4-2-EnzR cells (J). (I) Representative PI-stained histograms. Data are shown as mean ± SEM (n = 3).

Figure S2 Effects of knockdown or overexpression of UHRF1 on neuroendocrine markers and NSC232003 treatment in C4-2-EnzR

A-B) RT-qPCR results of NSE, SYP and SOX2 mRNA levels following UHRF1 knockdown in LNCaP-EnzR and C4-2-EnzR. C) mRNA levels of UHRF1-related genes in C4-2-EnzR 48 hours after 5μM NSC232003 treatment. D) Proliferation of C4-2-EnzR cells after 5μM NSC232003 treatment. E-F) RT-qPCR results of mRNA levels of MYC，NMYC and ENO2 in LNCaP and C4-2 cells 14 days after transfection with the negative control or UHRF1 overexpression plasmid. G) Effects of UHRF1 knockdown and enzalutamide on the viability of LNCaP-EnzR cells. After transfection with vector or shUHRF1 plasmid, cells were re-inoculated, and cck8 was added to some cells every 24 hours and incubated for 2 hours, after which the absorbance at 450 nm was measured.

Figure S3 Analysis of RNA-seq results after UHRF1 knockdown

A) Column plot of differentially expressed genes in irradiated NC and UHRF1 knockdown LNCaP-EnzR cells ($P < 0.05$, $|log2FC|\geq1$). B-C) KEGG and GO enrichment of differentially expressed genes. D) The top 20 most significant gene sets in the GSEA analysis. E-F) GSEA results for the cell cycle and DNA replication gene sets. G) Correlation analysis between UHRF1, E2F family genes, and certain neuroendocrine-related transcription factors.

Figure S4 IHC staining of UHRF1 in prostate cancer tissues.

A) Immunohistochemical staining of UHRF1. B) Clinical information of the patient. C) Western Blot results of UHRF1 and ENO2 expression in two C4-2-EnzR xenograft samples.

Figure S5. UHRF1 expression correlates significantly with key neuroendocrine lineage-defining transcription factors.

(A) Heatmap showing the correlation between UHRF1 and various gene modules across different cell clusters in the GSE264573 dataset. UHRF1 shows a significant positive correlation with NEPC-associated transcription factor modules (ASCL1, INSM1, SOX2, etc.).

(B) UMAP feature plots illustrating the spatial co-expression of UHRF1 with neuroendocrine and stemness markers SOX2, ASCL1, INSM1, PEG10, and ONECUT2.

(C) Heatmap of the Pearson correlation matrix for the expression of UHRF1 and key neuroendocrine driver genes. Red indicates a strong positive correlation.

Figure S6. Application of the UHRF1-regulated gene signature to human prostate cancer datasets.

(A–C) Gene Set Enrichment Analysis (GSEA) of bulk RNA-seq data from shUHRF1-treated C4-2-EnzR cells showing trends toward increased AR signaling, stem cell differentiation, and MET pathway activity, with reduced EMT and stemness maintenance signatures. (D) Application of the UHRF1-regulated gene signature to the SU2C/PCF prostate cancer cohort. Each patient was assigned a UHRF1 signature

score (mean expression of upregulated genes minus downregulated genes). High UHRF1 signature scores were associated with enrichment of neuroendocrine-related transcriptional programs and poorer clinical outcomes.
