# Supplementary material for: UHRF1 regulates AR ubiquitination to promote the loss of AR signaling and enzalutamide resistance in progression of prostate cancer
Source: Cell Death Dis. 2026 Feb 27;17(1):286. doi: 10.1038/s41419-026-08511-9 (PMC13031396; doi:10.1038/s41419-026-08511-9)

Fig1G

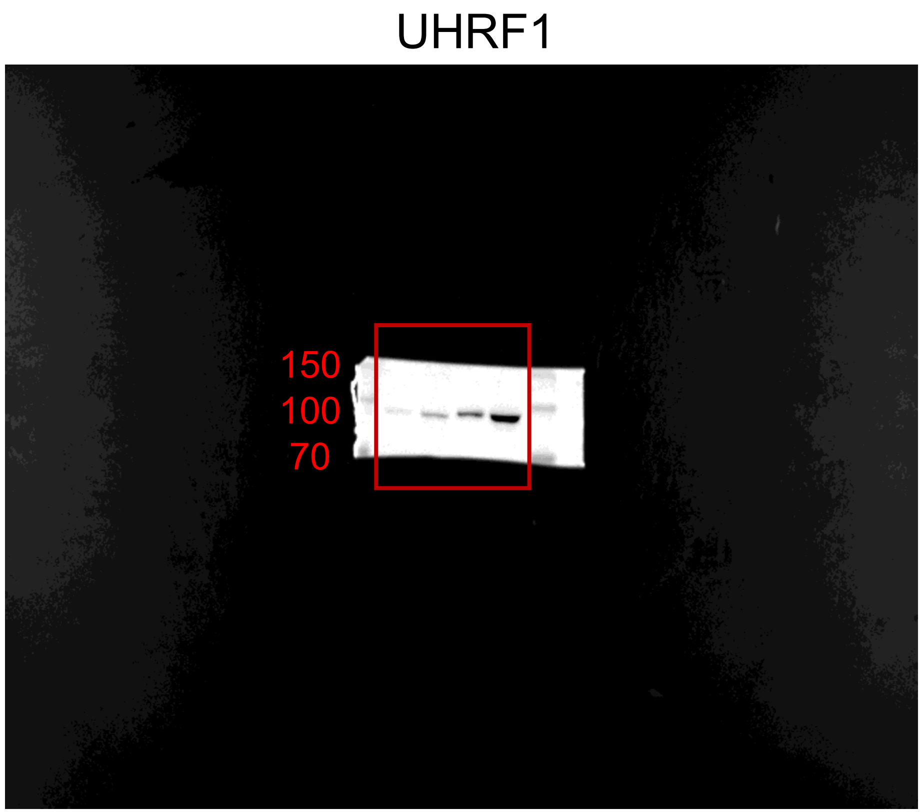

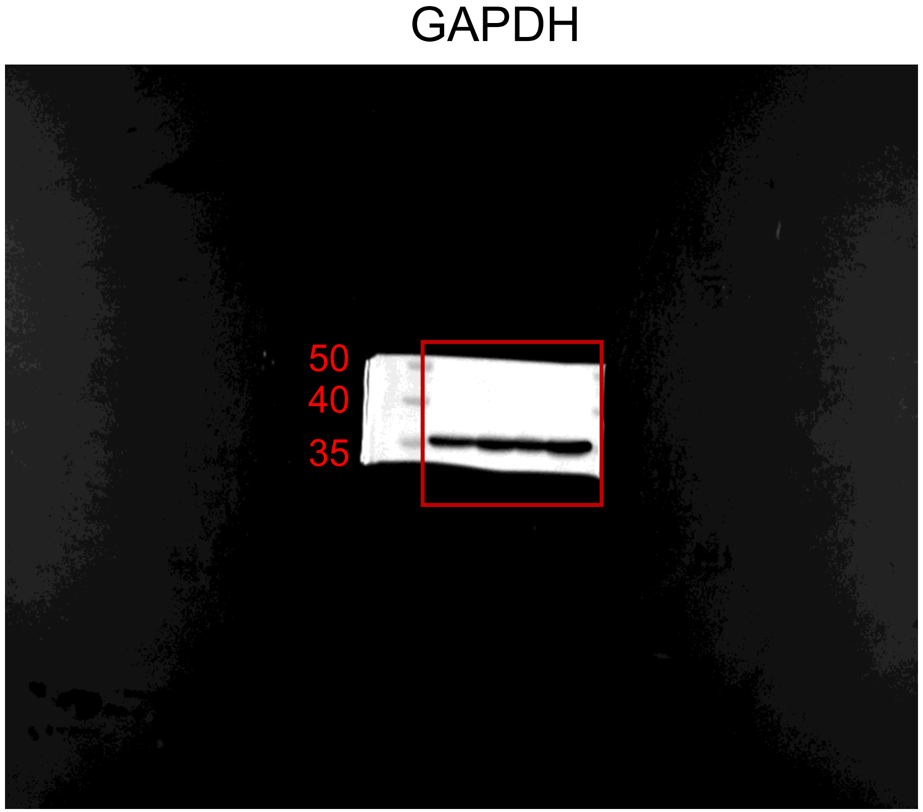

Fig2D

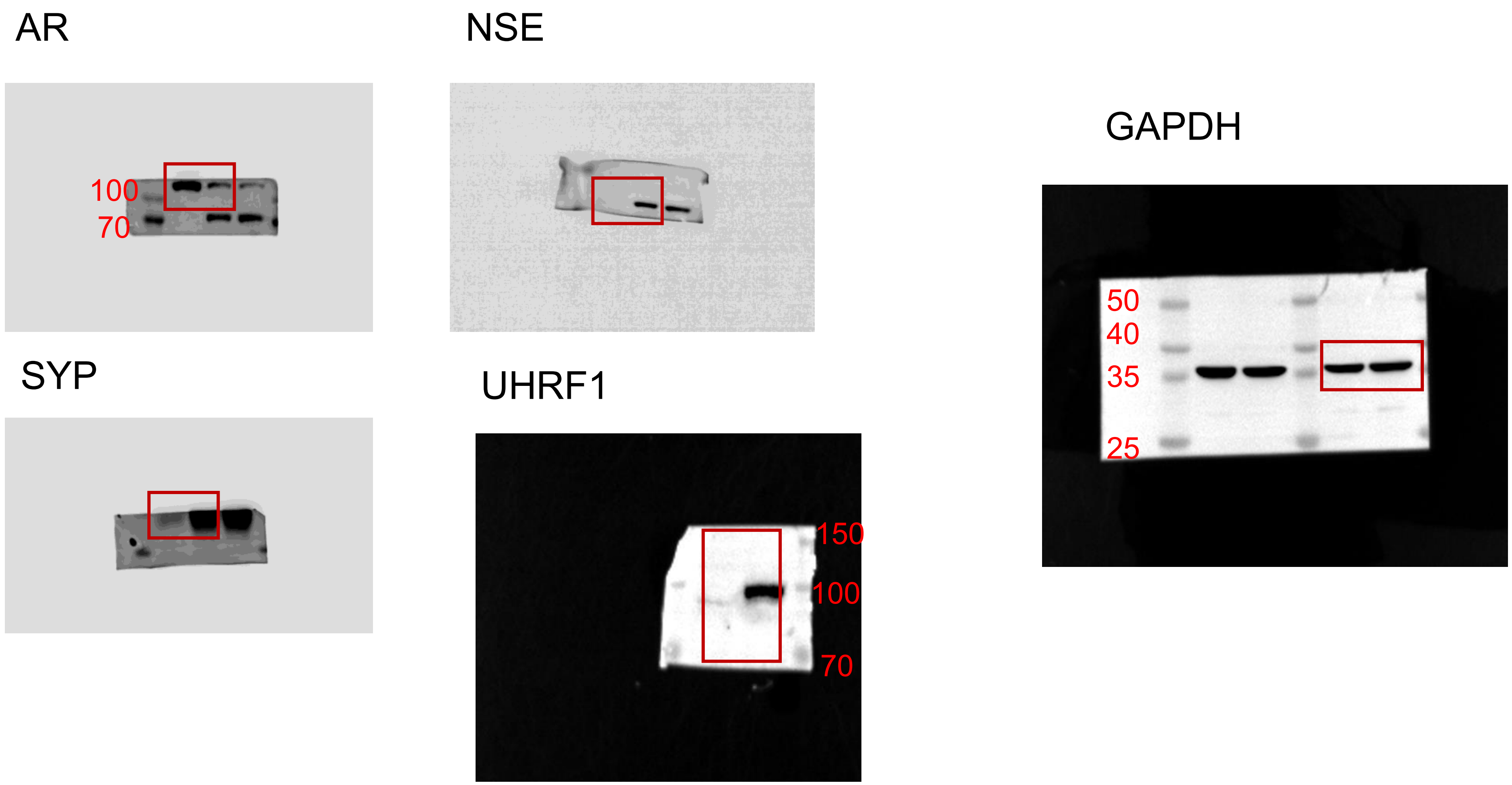

Fig2E

AR

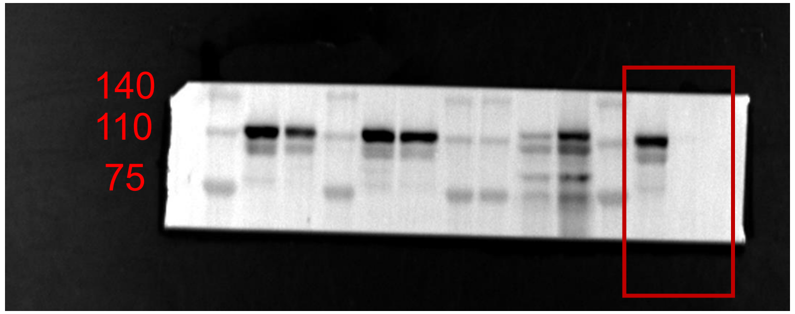

SYP

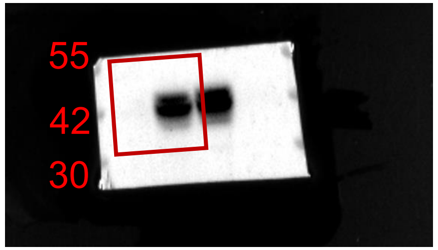

GAPDH

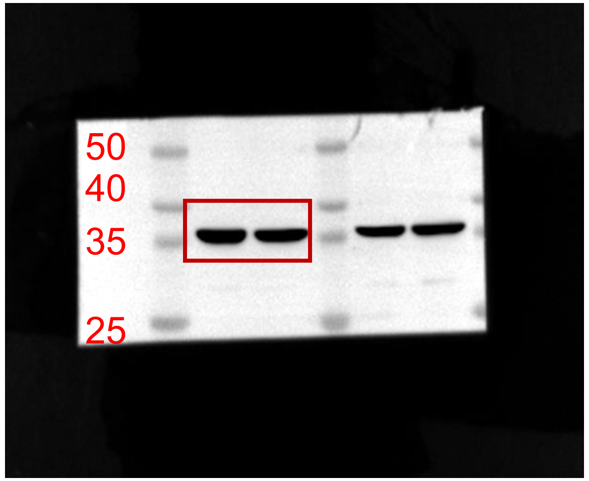

UHRF1

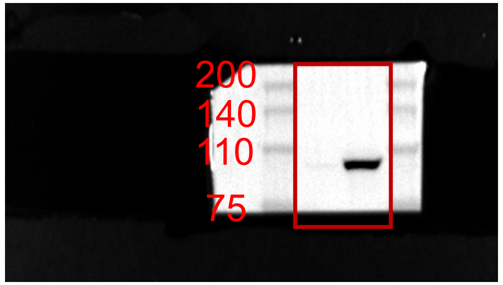

NSE

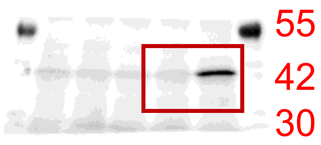

Fig3A

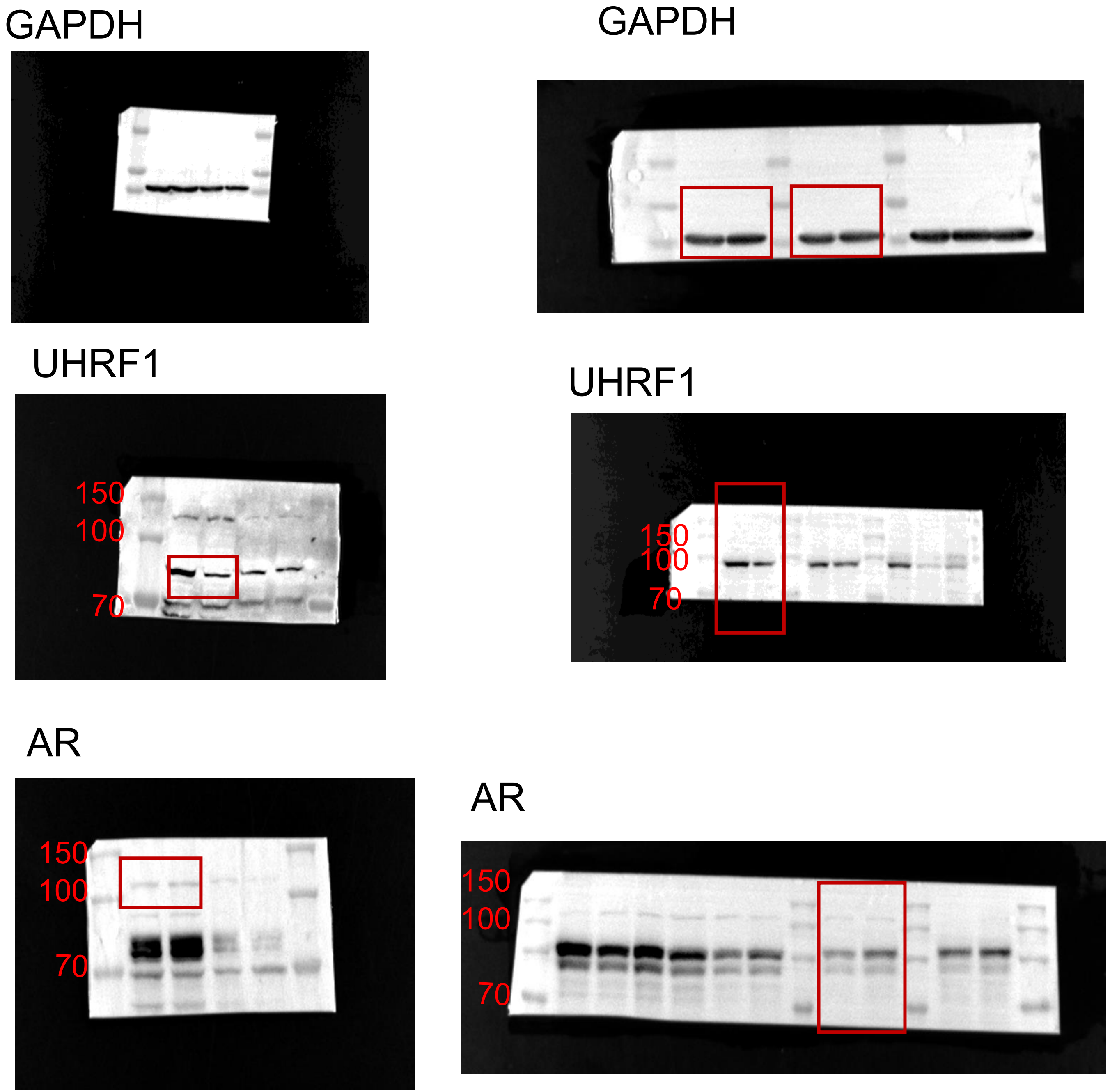
GAPDH
GAPDH
UHRF1
150
100
70
UHRF1
150
100
70
AR
150
100
70
AR
150
100
70

Fig3D

AR

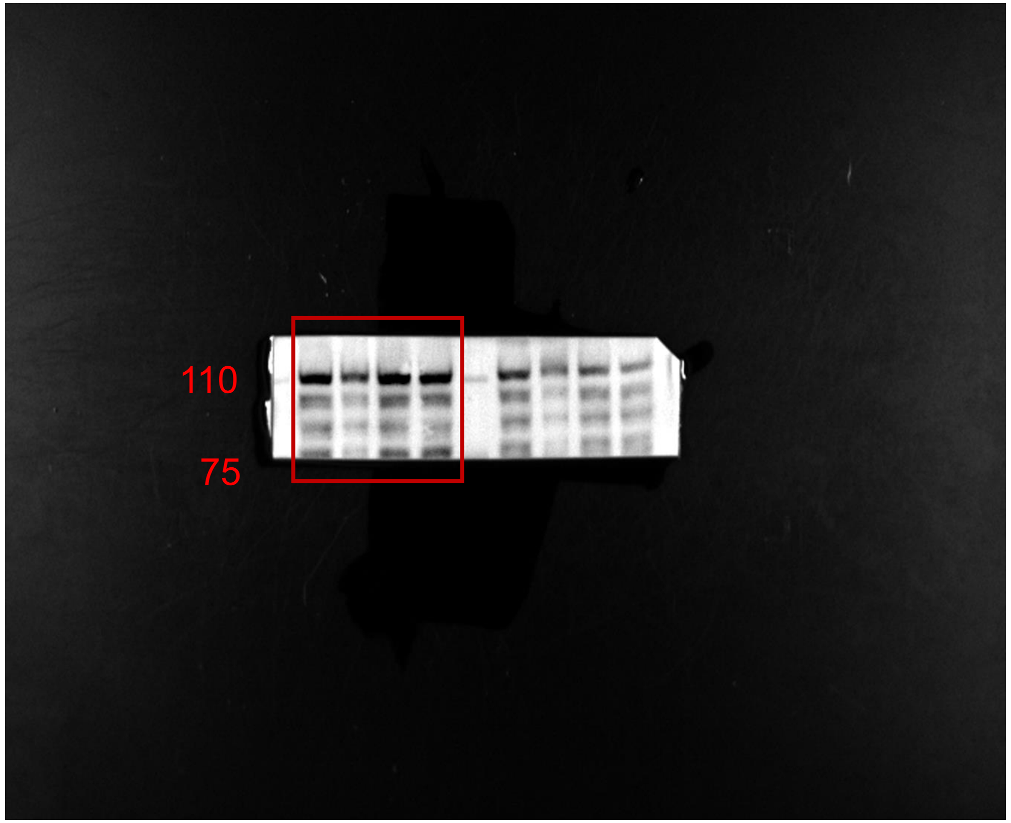

GAPDH

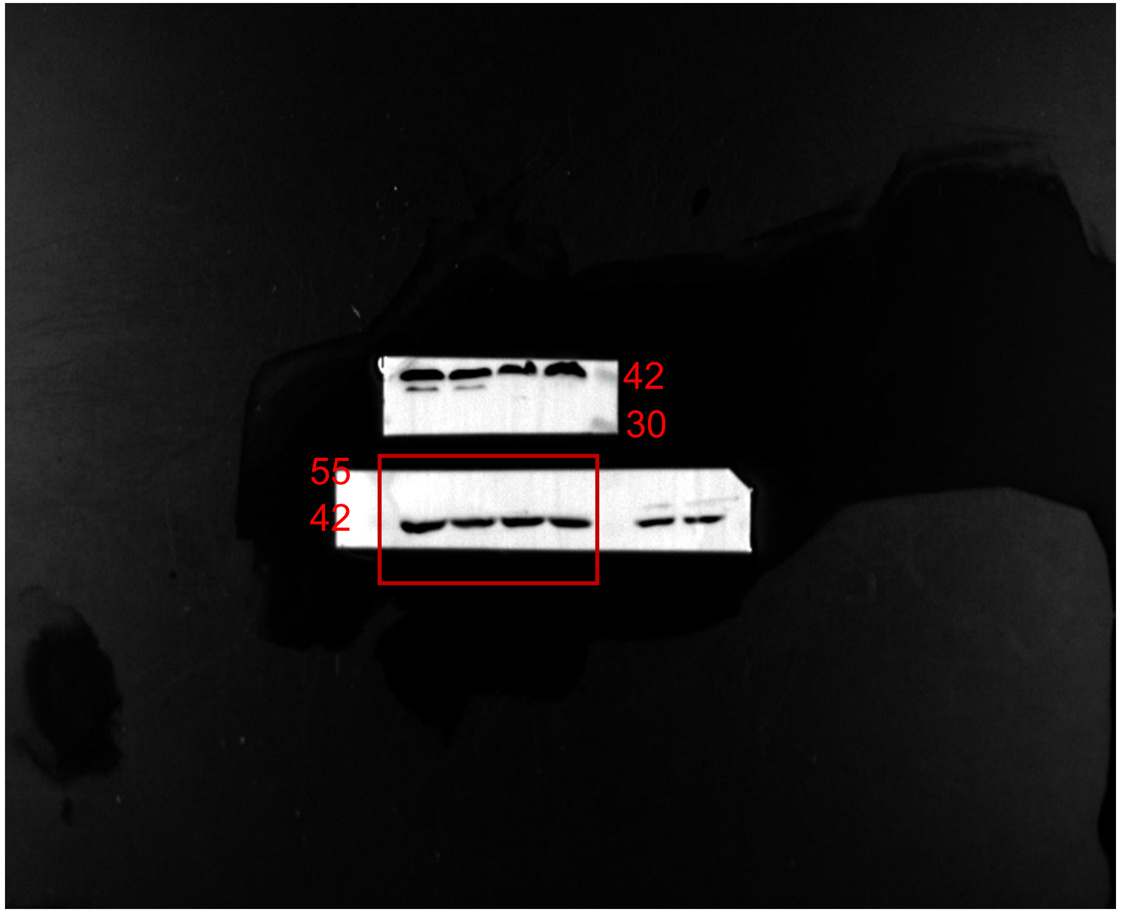

Fig3E

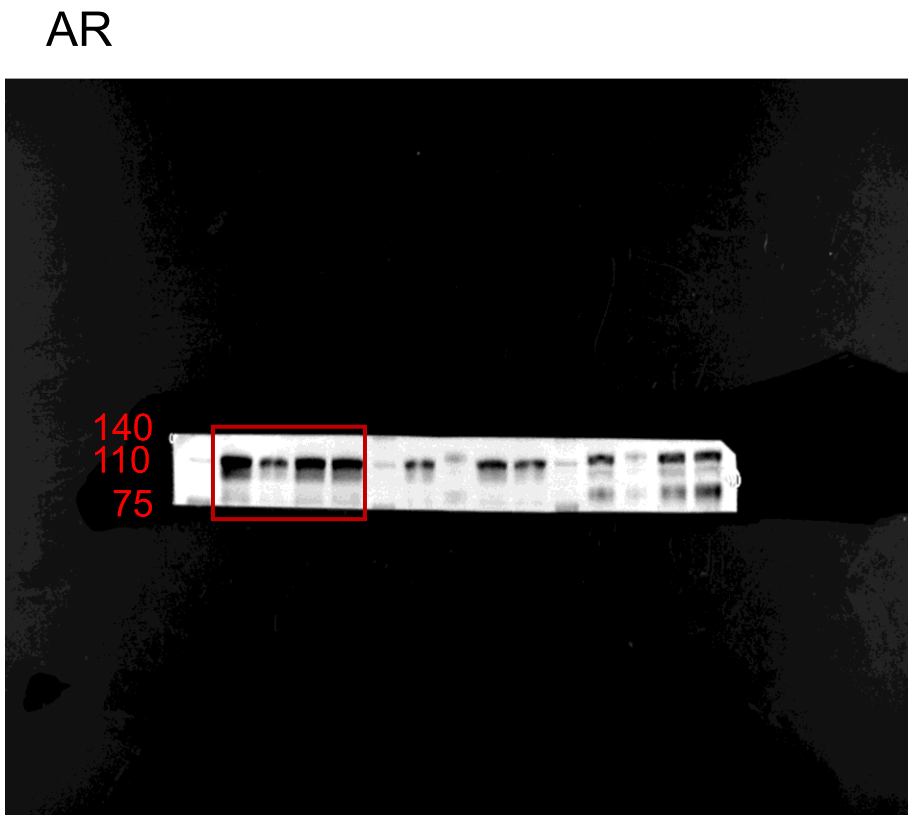

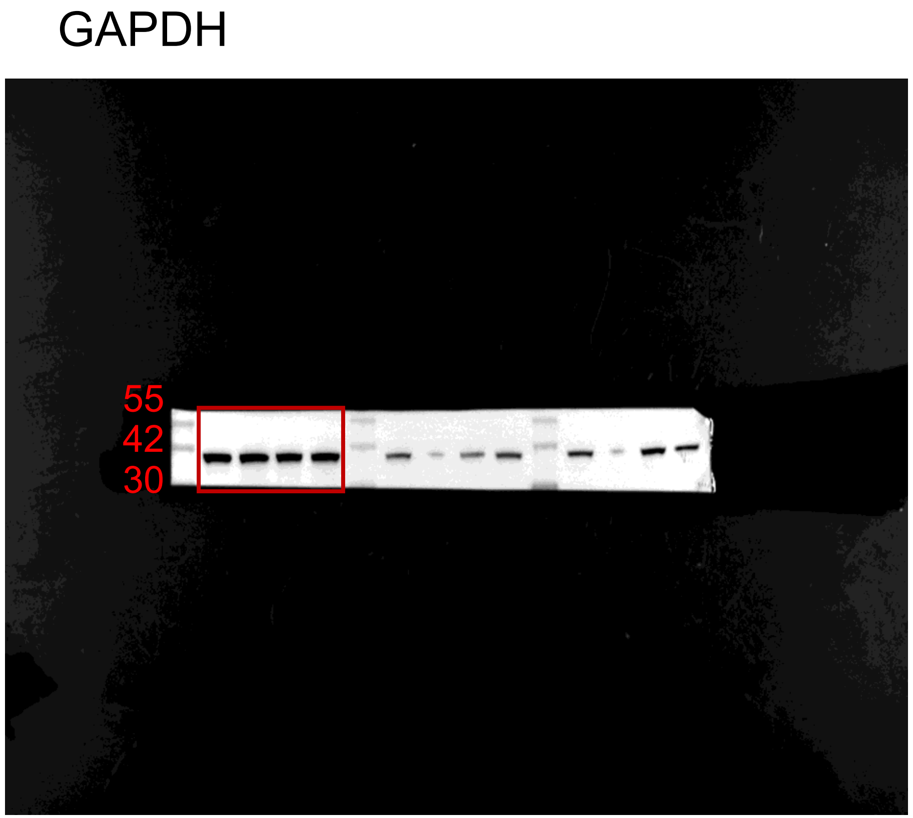

Fig3F

UHRF1

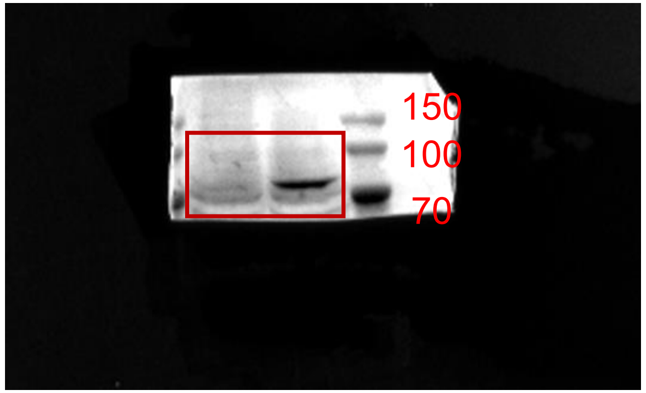

AR

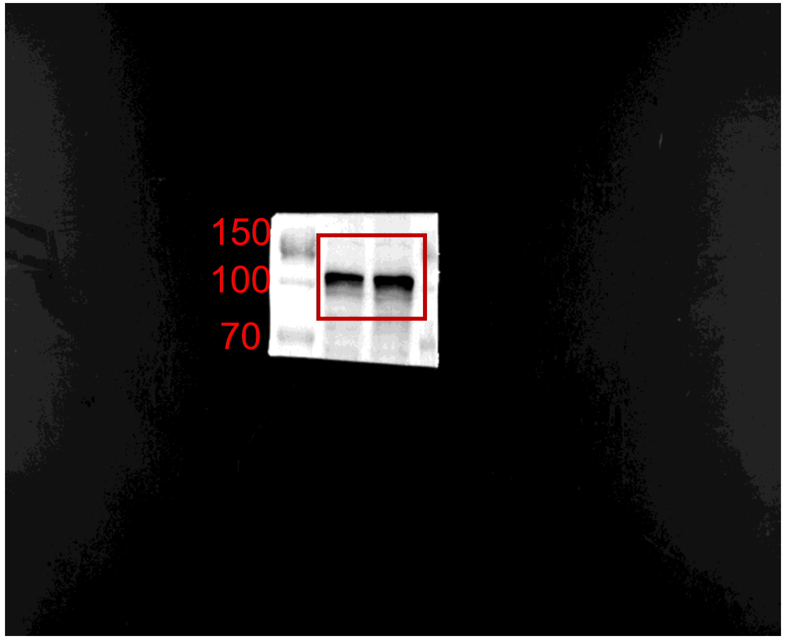

Ub

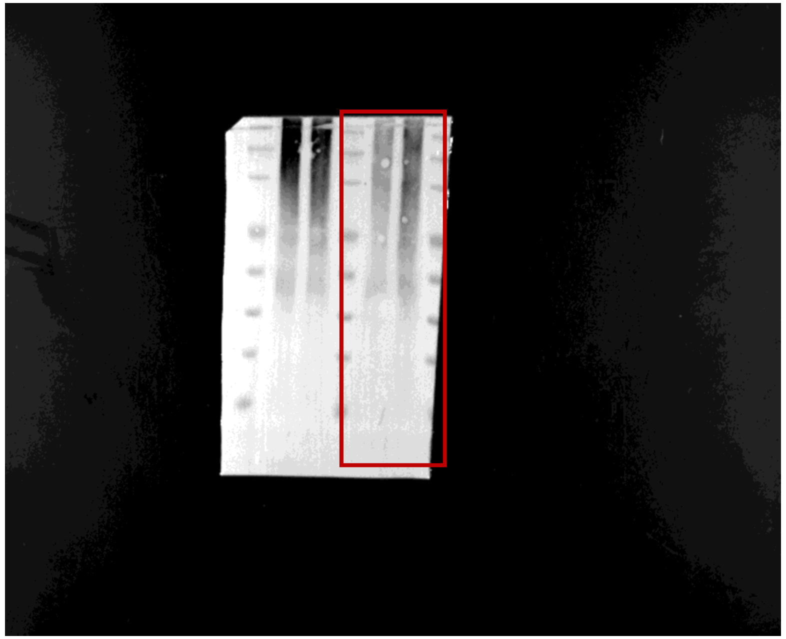

Fig3G

AR

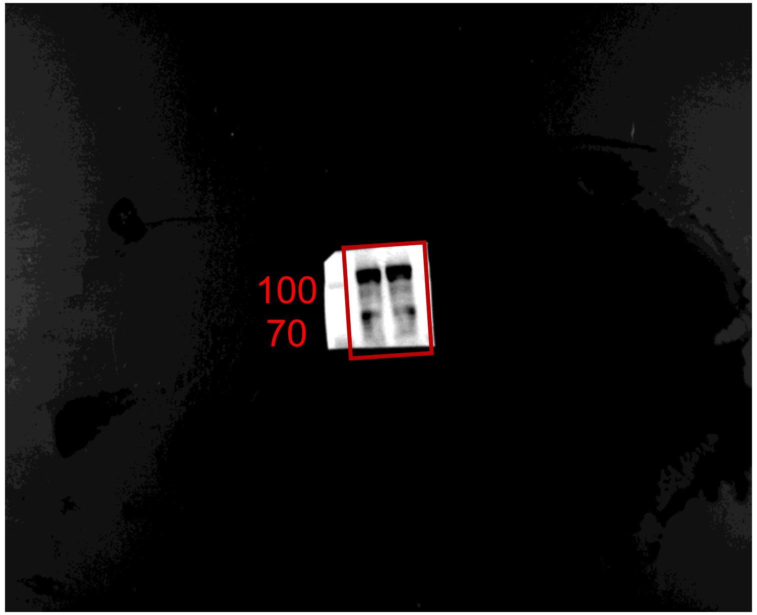

UHRF1

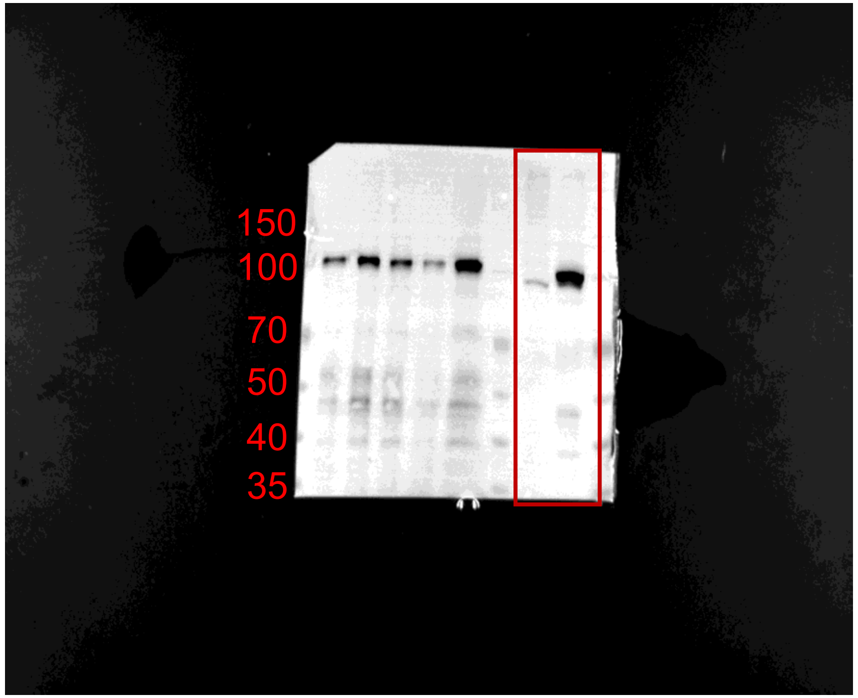

Ub

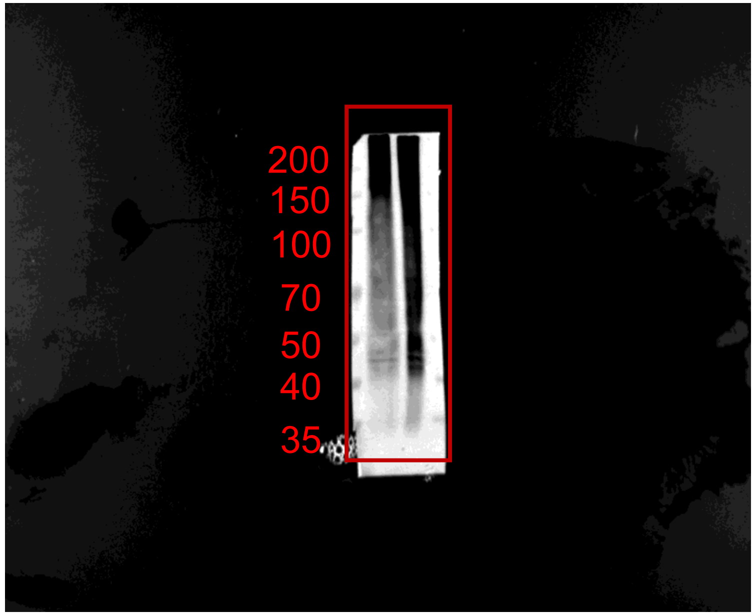

Fig3H

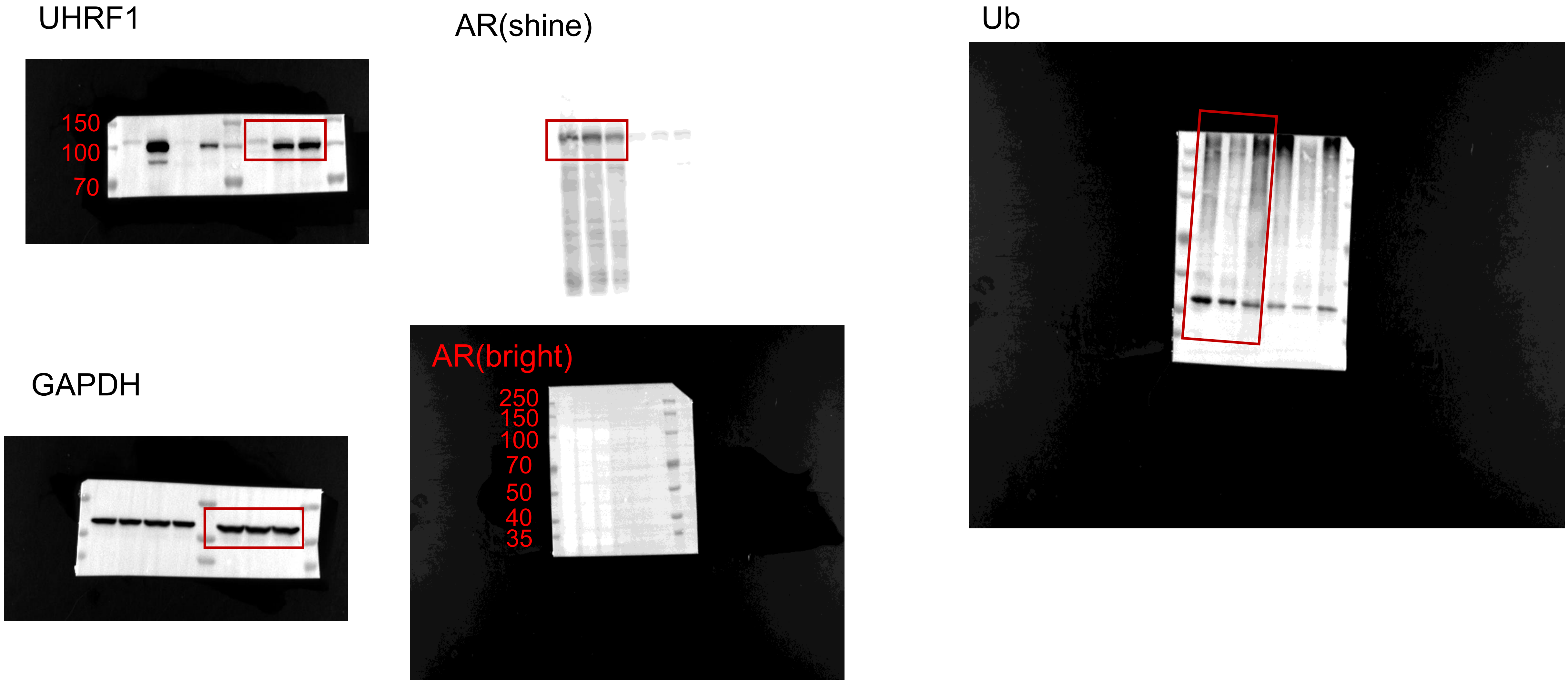
UHRF1
150
100
70
AR(shine)
Ub
GAPDH
AR(bright)
250
150
100
70
50
40
35

Fig3I

AR

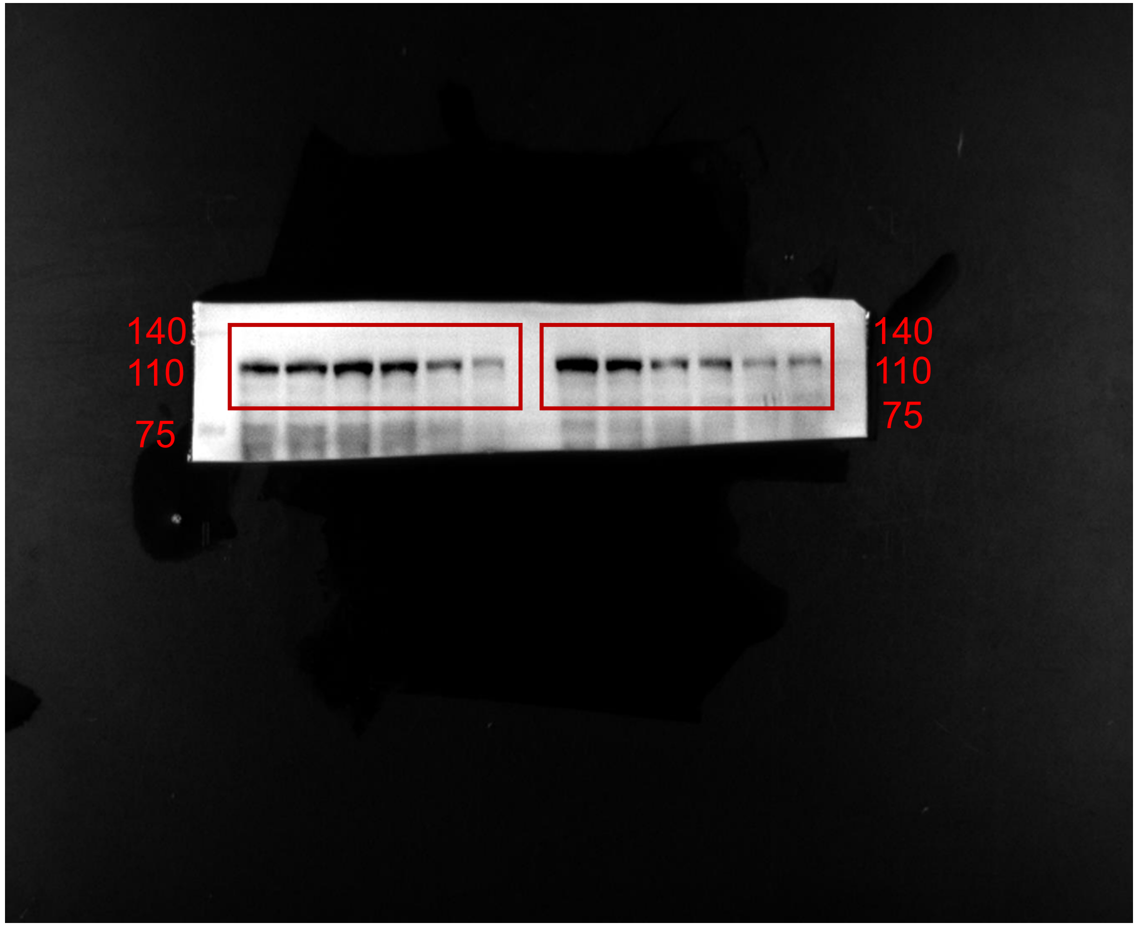

GAPDH

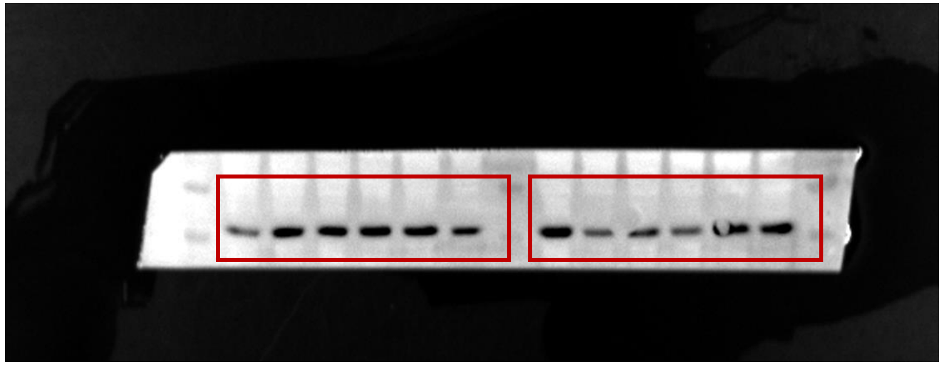

Fig3J

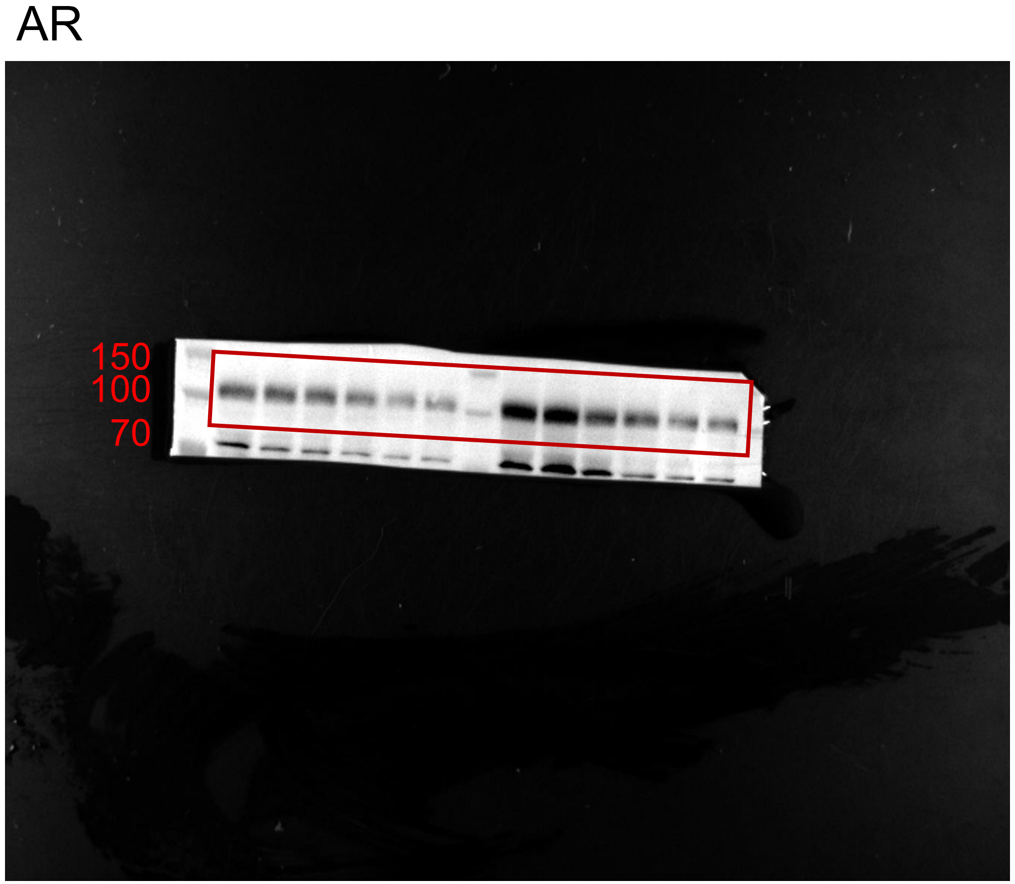

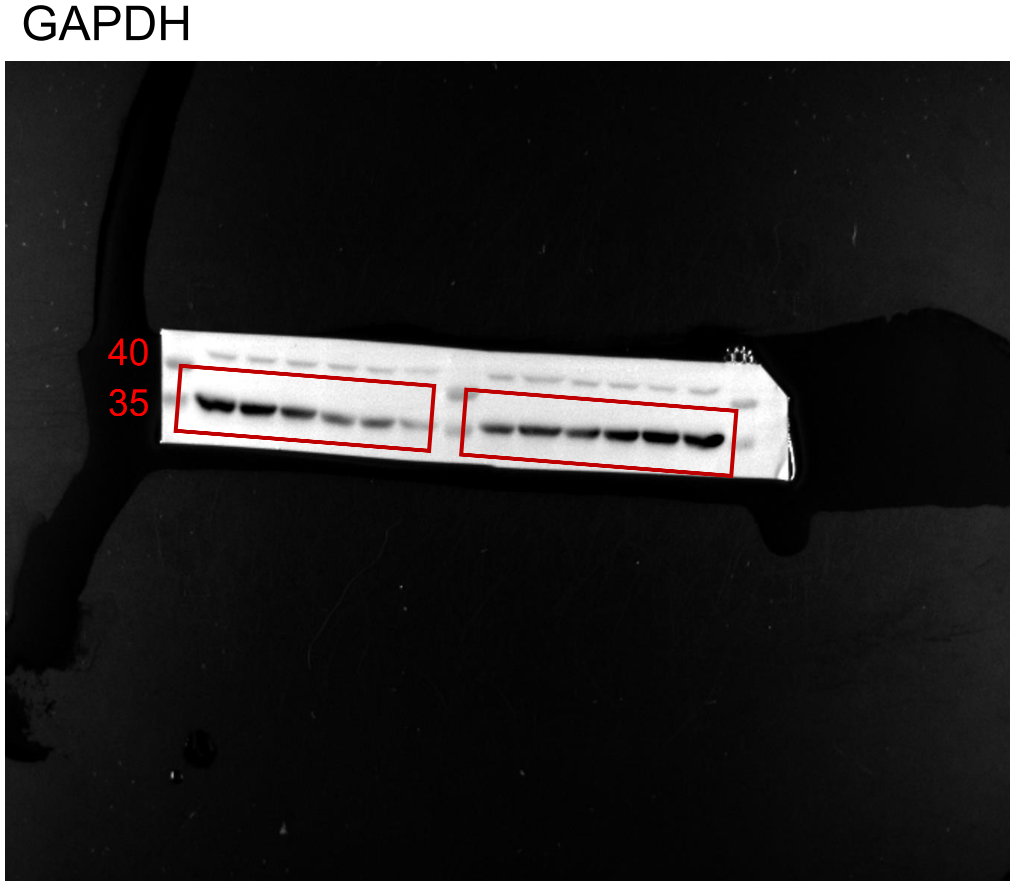

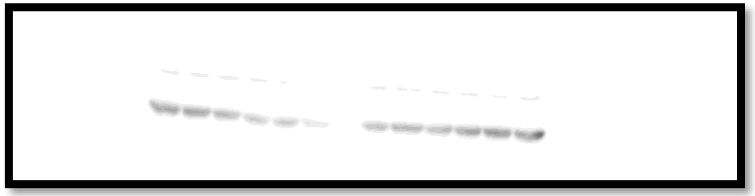

Fig3M

AR

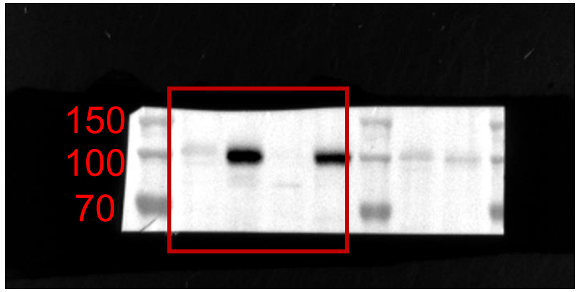

UHRF1

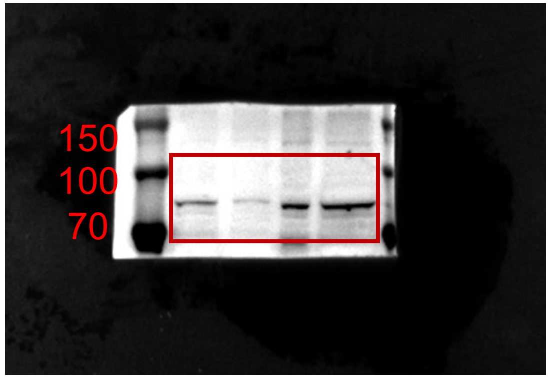

β-Actin

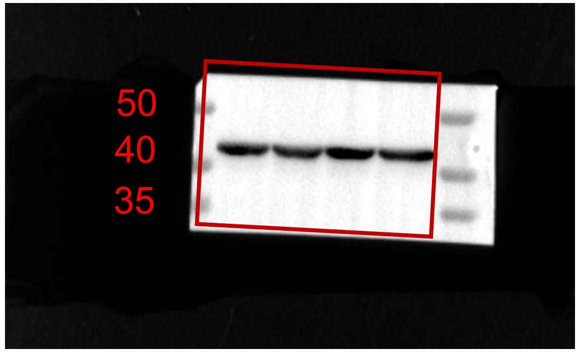

AR

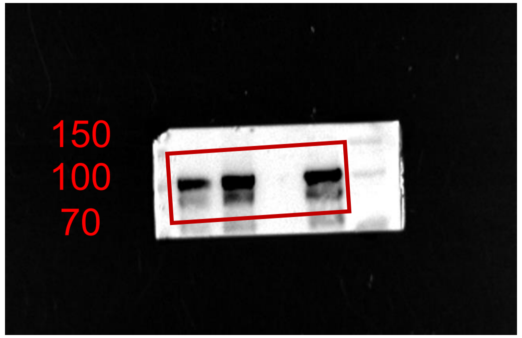

UHRF1

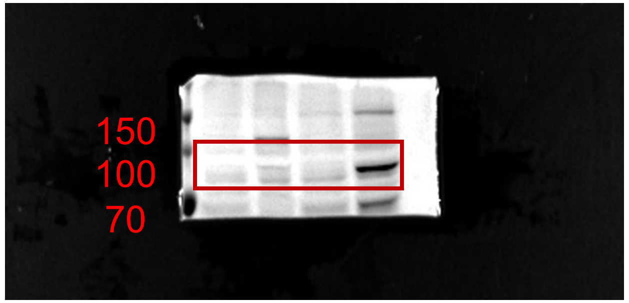

Fig3N

AR

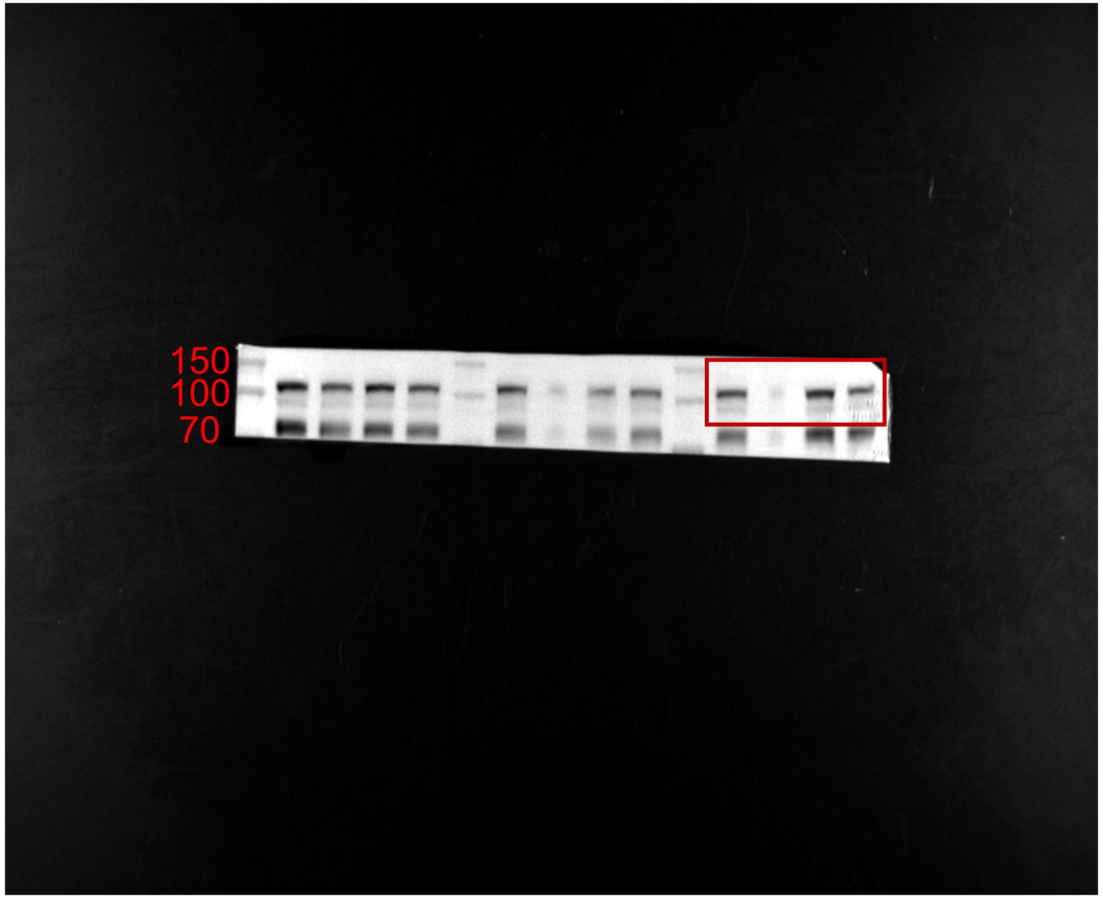

UHRF1

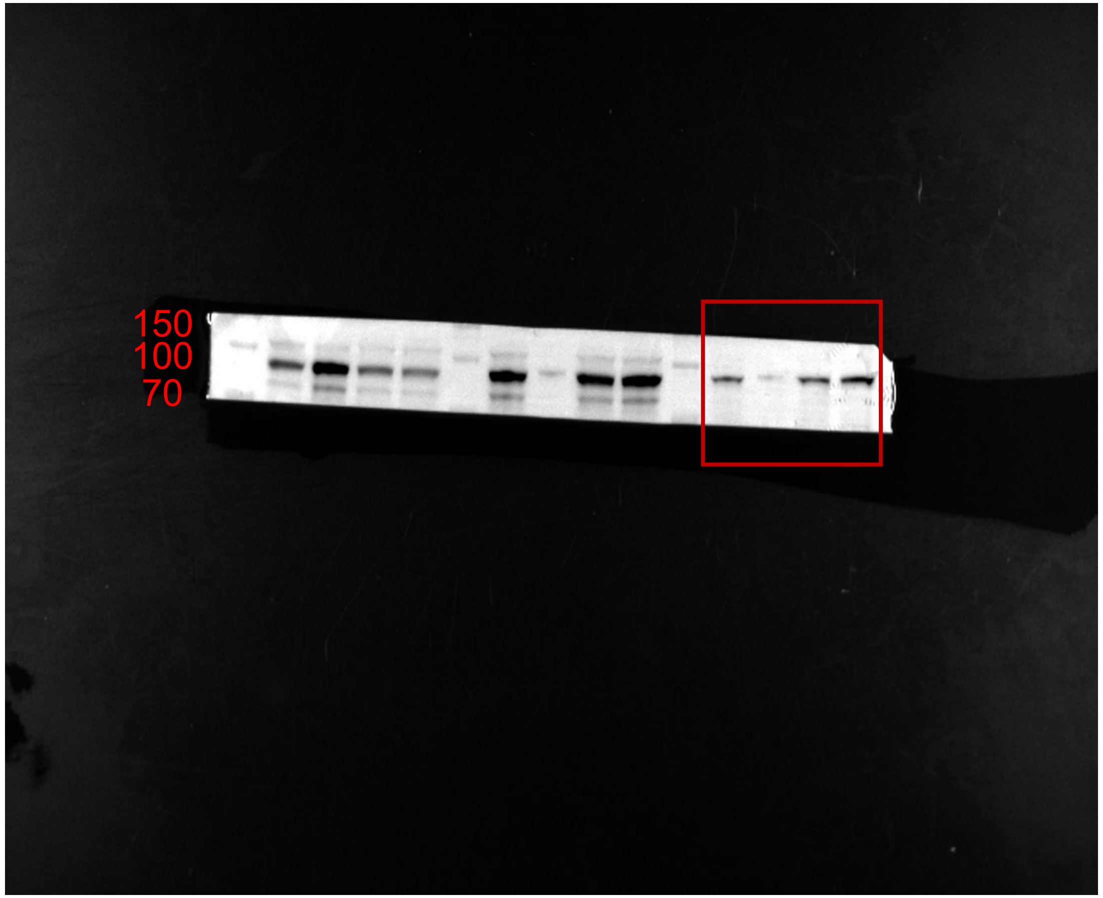

Fig3O

AR

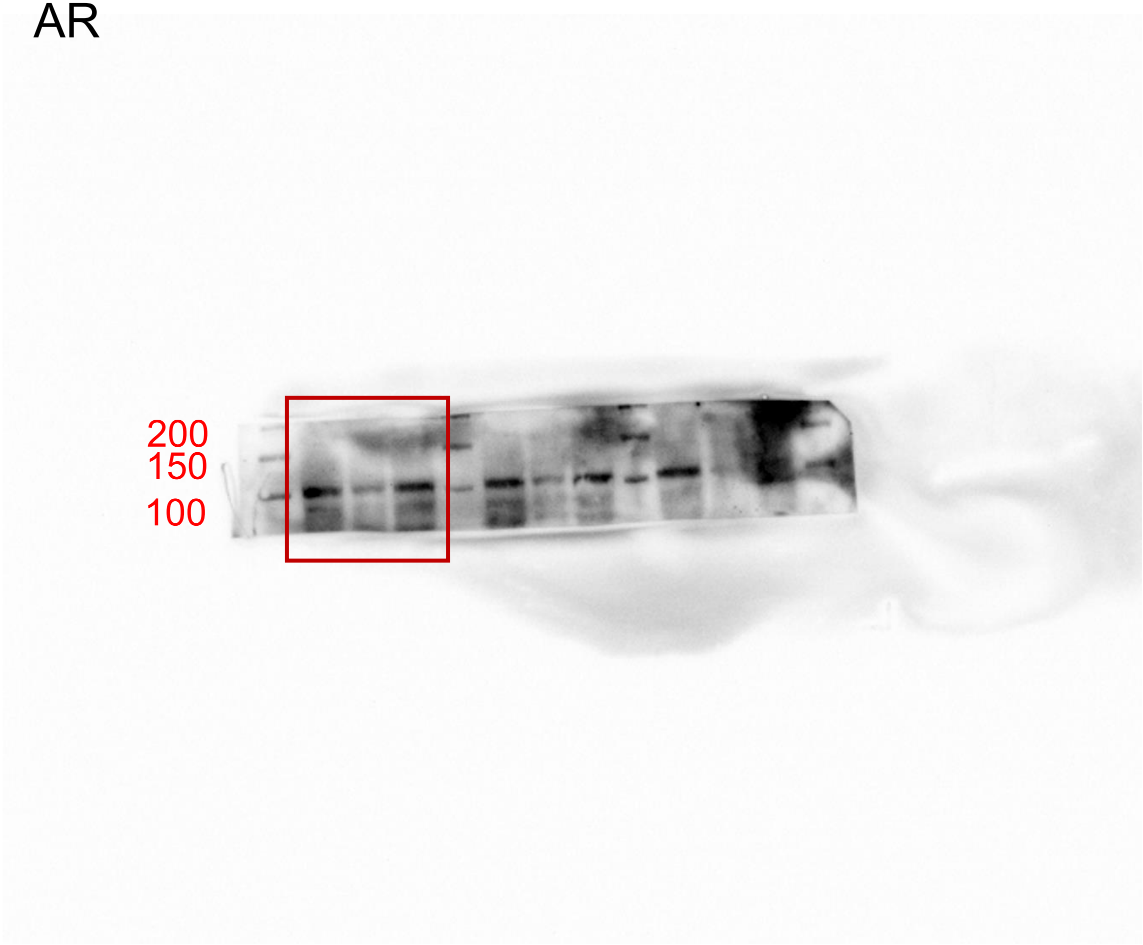

GAPDH

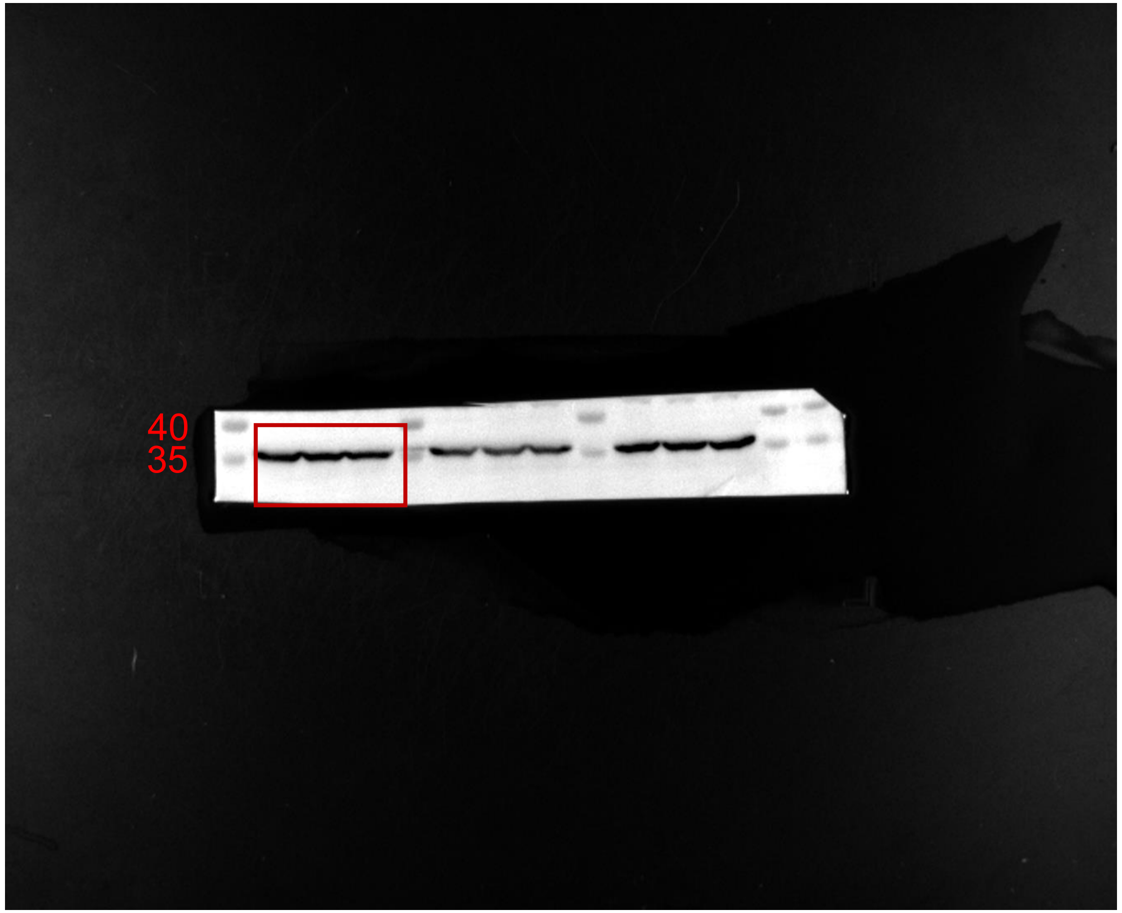

Fig3P

AR

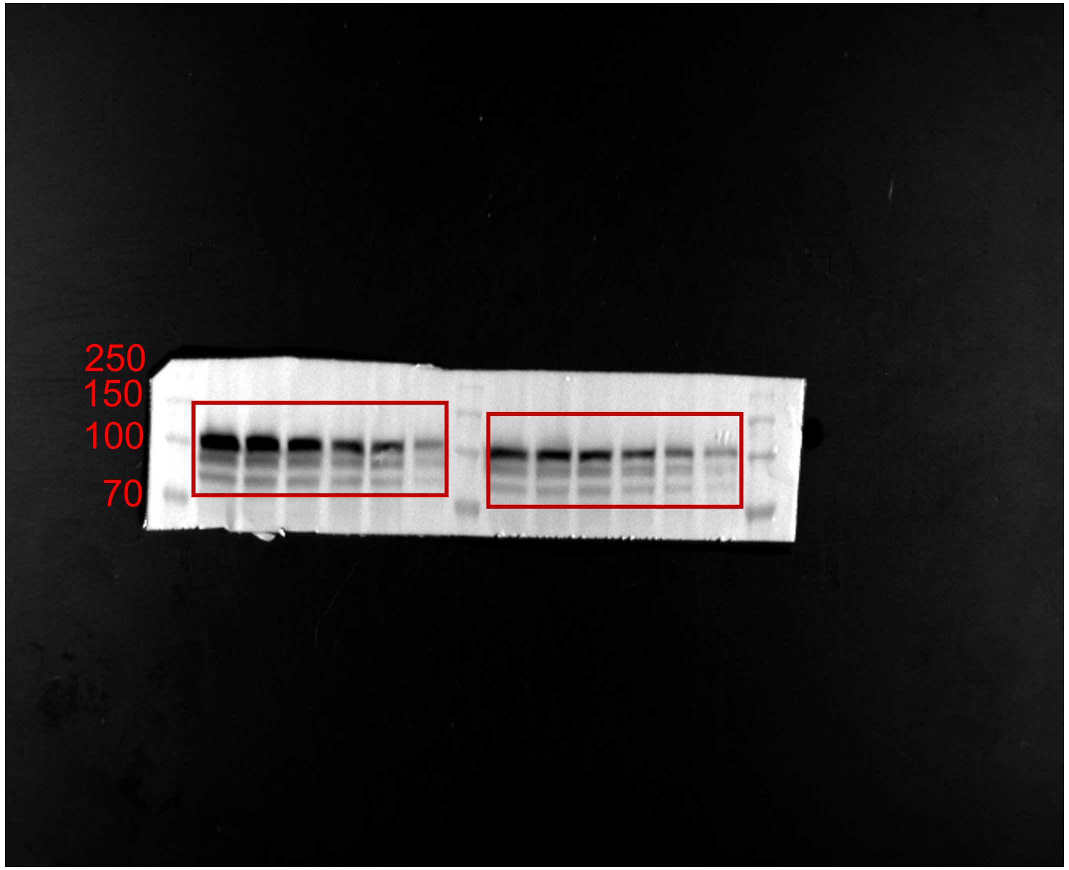

GAPDH

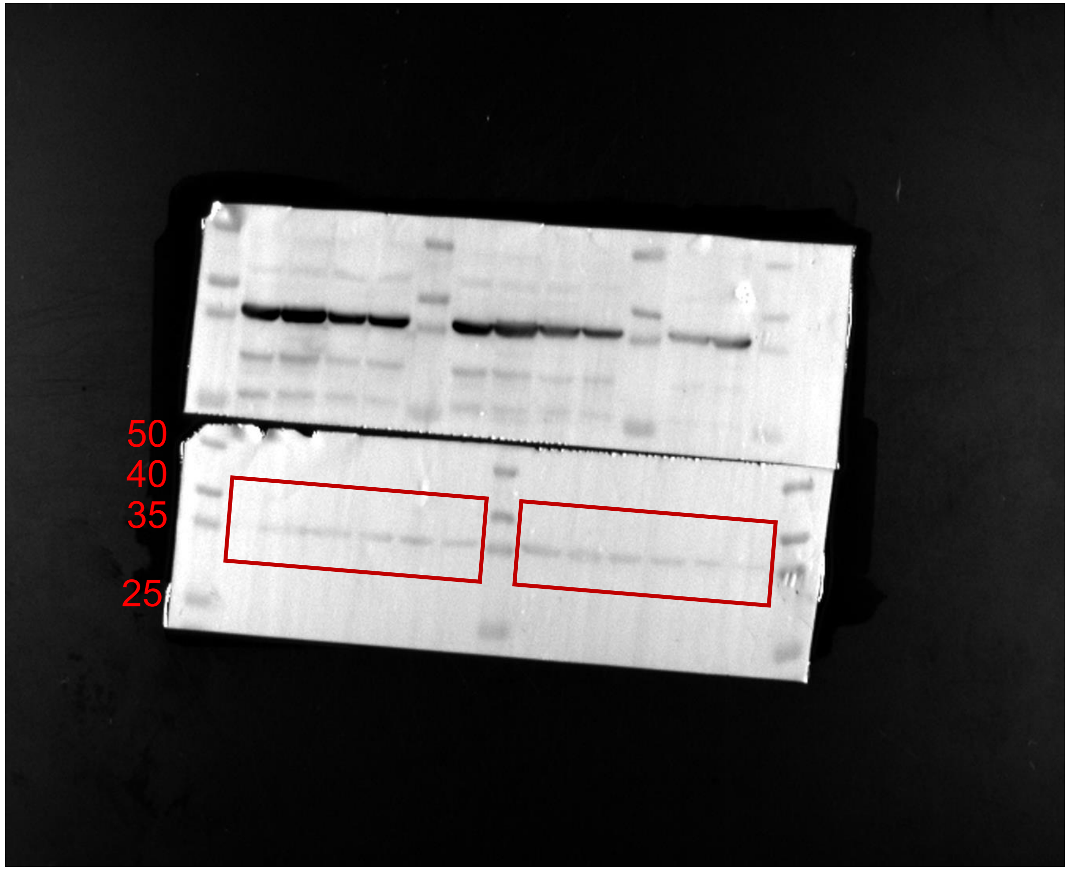

FigS4C

GAPDH

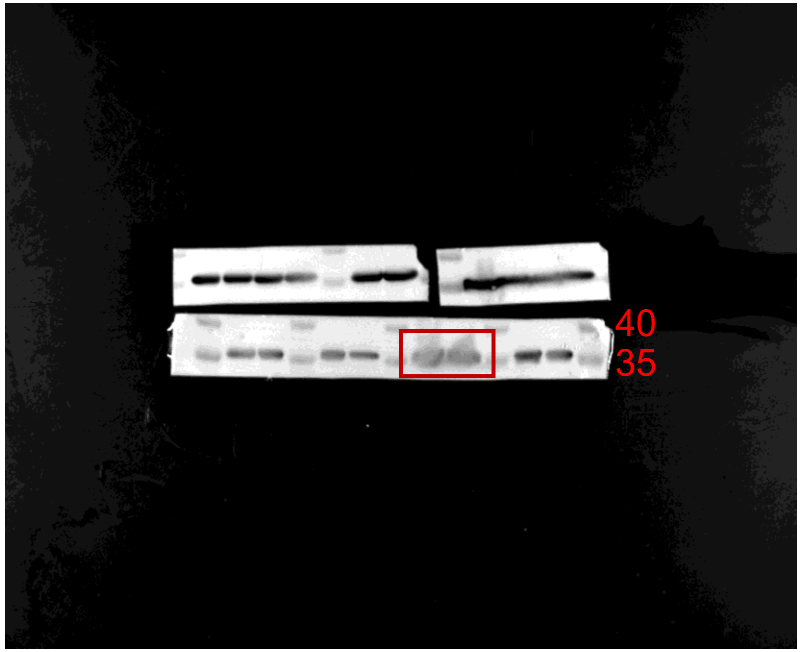

UHRF1

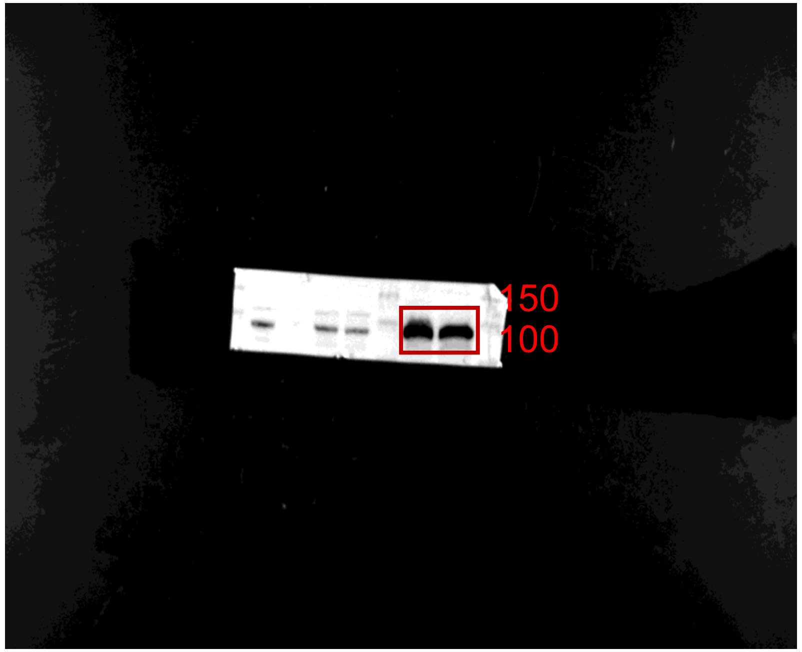

ENO2

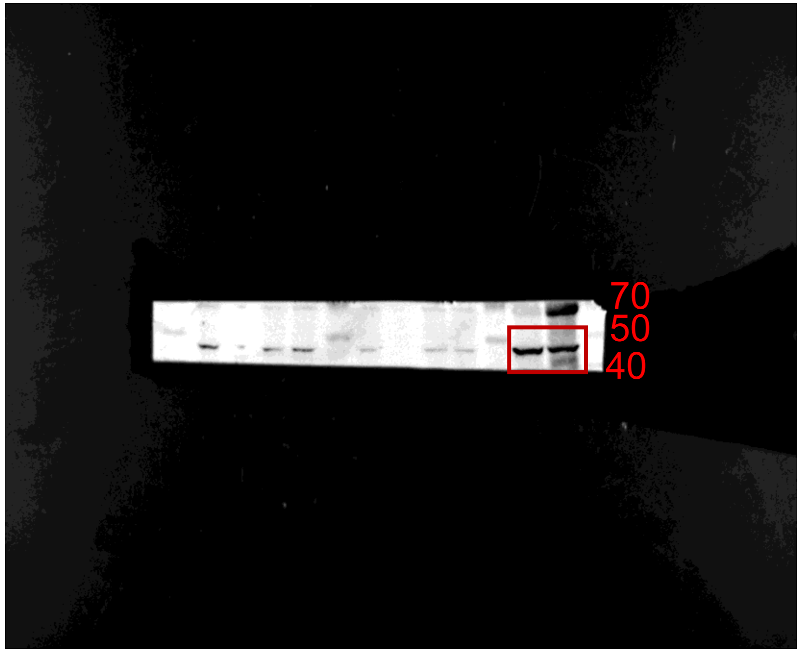

Supplement: Supplementary file 11 — Raw Data of Western Blot [file 41419_2026_8511_MOESM11_ESM.pdf]
